# Topological Progress Potential-Enhanced Continuous-Space Ant Colony Algorithm for Robot Path Planning

**DOI:** 10.3390/s26041264

**Published:** 2026-02-14

**Authors:** Guikun Dong, Feixiong Zhao, Jiaxiong Zhuo, Lei Zhou, Qiaoling Liu, Xiangjun Yang

**Affiliations:** School of Mechanical Engineering, Chengdu University, Chengdu 610106, China

**Keywords:** ant colony optimization algorithm, global path planning, continuous space, sector-based perception, elastic step size

## Abstract

To address the issues of traditional grid-based Ant Colony Optimization path planning in discretized continuous space—including limited direction freedom, lack of global topological guidance, and difficulty in balancing path smoothness and safety margin—a topological progress potential-enhanced continuous-space ant colony path planning algorithm (TPP-CSACO) is proposed. TPP-CSACO discards grid-based expansion; instead, a perception circle centered on each ant is defined, movement is executed via a sector-based perception framework with probabilistic direction selection, and band-shaped decaying pheromones are deposited along the path. By coupling the global topological progress potential derived from the simplified probabilistic roadmap (PRM) with pheromones, a dual-field guidance mechanism is established to prevent local congestion. Combined with the explicit safety constraints of the signed distance field (SDF), an adaptive step size strategy that integrates elastic step size and frustration-induced temperature rise is introduced to enhance obstacle avoidance and search stability. Results from repeated experiments on multiscale constrained maps (conducted against six typical algorithms and the traditional ACO) show that compared with ACO, TPP-CSACO reduces the path length by up to 50.6% in the same environment, while achieving faster convergence and maintaining good search diversity. Although the path length increases slightly (by a maximum of 5.9%) compared with the shortest heuristic algorithms, the maximum turning angle is reduced by 75% to 93%, and a 100% success rate and zero safety violations are realized. This indicates that TPP-CSACO has achieved a relatively stable balance among safety, smoothness, and global search capability.

## 1. Introduction

Path planning algorithms are a core technology in robotics [1]. They are designed to find an optimal or feasible path for robots from the starting point to the destination within their operational environments, while avoiding obstacles and satisfying specific constraints (e.g., minimum planning time, lowest energy consumption, and shortest path) [2]. Currently, path planning algorithms have been applied across various industries and domains, including agriculture [3], domestic services [4], military applications [5], and special equipment [6]. Extensive research has been conducted on path planning algorithms by researchers. Path planning algorithms are mainly classified into three main categories: classical algorithms, intelligent learning algorithms, and bio-inspired algorithms.

Classical path planning algorithms were first proposed and applied to deterministic environments, yielding results that are easily observable [7]. They mainly include cell decomposition (CD) [8], Rapidly-exploring Random Tree (RRT) [9], Dijkstra’s algorithm [10], A* algorithm [11], artificial potential field (APF) [12], and probabilistic roadmap (PRM) [13], among others. PRM was proposed by Overmars et al. in the early 1990s [14]. Its core principle is to perform random sampling in the configuration space and connect collision-free configurations to construct a roadmap, converting the search in complex continuous spaces into a discrete graph search. It is particularly suitable for path planning of high-degree-of-freedom robots and possesses strong probabilistic completeness and high multi-query efficiency [15,16]. Both PRM and RRT fall into the category of sampling-based methods: the former first constructs a static probabilistic roadmap before executing queries, while the latter gradually expands the path via a random tree structure [17]. A hierarchical path planner was proposed by Fan et al.: the upper layer utilizes PRM to rapidly generate a global guiding path, while the lower layer optimizes the path in segments through the Deep Deterministic Policy Gradient, which shortens the path length and enhances smoothness while satisfying the nonholonomic constraints of vehicles [18]. An incremental sampling strategy was adopted by Xu et al. to continuously construct and maintain PRM, enabling efficient and safe exploration of unmanned aerial vehicles (UAVs) in dynamic environments, which significantly reduces the total exploration time and computational cost [19]. To address the demand for online collision-free planning of 15-degree-of-freedom gantry welding robots in ship manufacturing, Zhou et al. proposed an improved Lazy-PRM algorithm that integrates rule guidance and a repulsive field (RR-Lazy-PRM). This algorithm notably reduces the collision risk between the robot, workpieces, and its own joints; additionally, it enables the collaborative operation of dual manipulators, precisely meeting the dual requirements of path accuracy and operational efficiency in welding scenarios [20].

Intelligent learning algorithms are generally defined as a category of artificial intelligence (AI) [21] methods that are characterized by data-driven or interaction-based learning and that, supported by modern computational resources, automatically acquire knowledge, decision-making strategies, or mapping relationships. Representative approaches include artificial neural network (ANN) [22], fuzzy logic (FL) [23], and reinforcement learning (RL) [24], among others. A CBNNP algorithm integrating bio-inspired neural networks and an ocean current compensation mechanism was proposed by Zhu et al., which allows unmanned underwater vehicles (UUVs) to stably track the optimal path in environments with different current directions, flow velocities, and obstacles [25]. The Artificial Bee Colony (ABC) algorithm was adopted by Aliskan et al. to optimize the output membership function of the fuzzy logic controller (FLC). Based on the Integral of Absolute Error (IAE) and Integral of Time-weighted Absolute Error (ITAE) indices, the FLC-IAE and FLC-ITAE controllers were designed, and the effectiveness of the metaheuristic algorithm in parameter tuning for automated guided vehicle (AGV) steering control was verified [26]. The potential field was encoded as a neural field by Fareh et al. to realize fast path planning for the leader, significantly improving the execution speed and stability of the system [27].

Bio-inspired algorithms are inspired by the behaviors of individual organisms, colony behaviors, and specific physiological functions (e.g., foraging, social interaction, escape, etc.) [28]. They mainly include the genetic algorithm (GA) [29], particle swarm optimization (PSO) [30], Grey Wolf Optimizer (GWO) [31], and Ant Colony Optimization (ACO) [32], among others. ACO simulates the foraging behavior of ants, searching for the optimal path via the positive feedback mechanism of pheromones [33]. It exhibits strong robustness and excellent parallelism, yet has inherent drawbacks such as slow convergence and a tendency to get trapped in local optima [34,35]. A multi-strategy adaptive ant colony algorithm was proposed by Cui et al., which integrates four improvements: direction guidance, adaptive heuristic function, deterministic state transition, and non-uniform pheromone initialization. This algorithm significantly enhances convergence speed and path quality and outperforms the traditional A* and ACO algorithms in various complex environments [36]. For orchard mowers, a BL-ACO path planning and GO-SMC tracking control scheme was proposed by Liu et al. The BL-ACO optimizes the operation path by improving the pheromone update mechanism and adopting a two-layer optimization strategy, while the GO-SMC designs a control law based on the kinematic model to ensure tracking stability [37]. A goose optimization-based ant colony algorithm was proposed by Sheng et al. In traveling salesman problem (TSP) and AGV scenarios, this algorithm achieves a more optimal path length and significantly improved convergence efficiency and demonstrates stronger adaptability in dynamic environments such as tobacco workshops [38]. A multi-strategy genetic ant colony algorithm was proposed by Li et al. It can stably obtain the optimal path in multiscale grid environments and can efficiently handle obstacle avoidance and path planning in dynamic obstacle environments [39]. An improved Q-evaluation Ant Colony Optimization algorithm was proposed by Li et al. This algorithm significantly improves path quality and convergence speed in small- and medium-scale maps; even in large-scale environments, it can maintain a fast solution speed, effectively enhancing the stability and real-time performance of path planning [40]. In 2008, Socha and Dorigo extended the Ant Colony Optimization framework to continuous domains and proposed the Ant Colony Optimization for Continuous Domains (ACOR) algorithm, which employs a solution archive to dynamically generate the Gaussian kernel probability density function and guide ants to perform sampling searches in the continuous space [41]. Based on ACOR, Niu et al. proposed an Ant Colony Optimization with an improved state transition probability, a random-walk strategy, and an adaptive waypoints-repair method, which is integrated with the dynamic window approach to enhance the global search efficiency and dynamic obstacle avoidance capabilities for UAV 3D path planning [42]. Wang et al. proposed a reinforcement-learning-driven multi-strategy continuous Ant Colony Optimization algorithm that demonstrates superior performance in addressing the multi-UAV path planning problem in complex three-dimensional continuous environments [43].

Current research on ACO-based path planning algorithms can be broadly classified into two categories: grid-based discretization methods and continuous-domain methods. Although grid-based discretization methods feature straightforward implementation, the discretized environmental model inherently restricts the motion flexibility and safety of robots, and the generated path morphology is constrained by the grid structure with fixed step sizes and orientations. Such discretization is usually accompanied by simplifying robot dimensions and binarizing obstacles, resulting in a lack of fine-grained characterization of continuous distance fields and safety margins; thus, an optimal trade-off between path smoothness and wall-proximity risk cannot be achieved. Continuous-domain methods are free of the limitations of grid resolution but cannot accurately model the geometric constraints of obstacles. In addition, neither category has sufficient awareness of the environment’s global topological connectivity. Therefore, in multi-channel, maze-like scenarios, the ant colony algorithm is prone to becoming trapped in local optima that are geometrically adjacent but topologically infeasible. To address the above limitations, this paper proposes a topological progress potential-enhanced continuous-space ant colony algorithm for path planning (TPP-CSACO), whose core designs are as follows: (1) A sector-based perception and probabilistic direction selection framework in continuous space is proposed. By adopting sector-based perception and multidirectional probabilistic selection in the continuous coordinate space to replace the traditional grid neighborhood expansion mode, the directional freedom of ants and the potential for smooth paths in static environments are improved. (2) An adaptive search strategy coupling elastic step size and frustration-driven temperature rise is proposed. By integrating adaptive step size scaling and frustration-driven temperature adjustment, the search granularity and exploration intensity are automatically adjusted according to the degree of local blockage, thereby enhancing the algorithm’s path-finding capability in complex environments. (3) An explicit safety margin modeling and dual constraint mechanism oriented to robot size is constructed, which effectively reduces wall-hugging paths and achieves a more reasonable trade-off between path length and path safety. (4) A planning framework based on PRM topological progress potential and nucleated pheromone corridors is proposed. By combining the topological progress potential constructed on the simplified PRM graph with nucleated pheromone deposition in continuous space, the global topological prior and local swarm experience are unified into the mobile decision-making process. The remainder of this paper is structured as follows. Section 2 presents the scheme for constructing the environment model. Section 3 describes the design of the TPP-CSACO. Section 4 elaborates on the process of the TPP-CSACO path planning algorithm. Section 5 conducts simulation experiments and corresponding analysis. Conclusions are drawn in Section 6.

## 2. Environment Model Construction

In mobile robot path planning, environment modeling serves as the foundation for safe navigation and simulation. In this paper, obstacles $O_{k}$ are represented by closed polygons composed of ordered vertices $\left\{V_{1},V_{2},\ldots ,V_{n}\right\}$. The set of K obstacles is expressed as Equation (1):

(1) $$\tilde{O}={⋃}_{k}^{K}O_{k}$$

**Figure 1:**
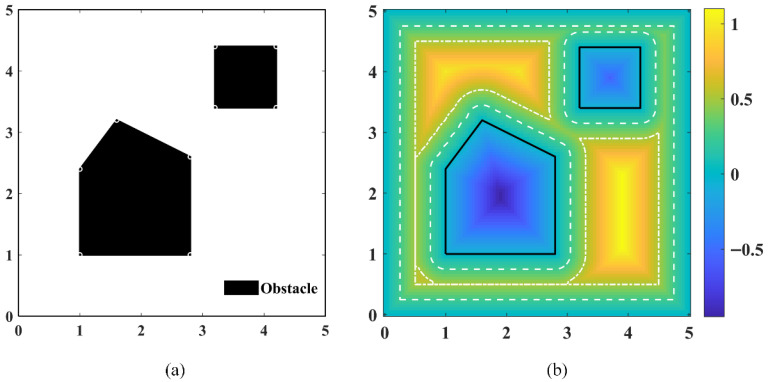
Construction of the operating environment. (**a**) Construction of obstacles. (**b**) Construction of the signed distance function (SDF) field.

This method can accurately depict the contours of complex obstacles, facilitating the subsequent calculation of the signed distance field (SDF). To quantify the collision state and safety margin of any point in the continuous space, the SDF is introduced: the SDF of an arbitrary point P=(x,y) in the space is defined as the signed distance from P to the nearest obstacle boundary $\partial \tilde{O}$, as given in Equation (2):

(2) $$SDF=\left\{\begin{array}{cc} -d(P,\partial \tilde{O}), & P\in \mathrm{int}(\tilde{O})\\ +d(P,\partial \tilde{O}), & otherwise \end{array}\right.$$

where d(P,∂Ω) denotes the Euclidean distance from the point P to the obstacle boundary. Figure 1a illustrates the construction of closed obstacles. As shown in Figure 1b, the interior of obstacles takes negative values, while the SDF of free space increases as the distance to the obstacles increases.

To ensure the spatial smoothness of SDF queries, bilinear interpolation is employed as the continuous query interface. For any query point, its corresponding interpolation grid cell is first determined. Let the coordinates of the four corner points of this grid cell be $Q_{11}=(x_{1},y_{1})$, $Q_{21}=(x_{2},y_{1})$, $Q_{12}=(x_{1},y_{2})$, and $Q_{22}=(x_{2},y_{2})$, with their respective SDF values denoted as $F_{11}$, $F_{21}$, $F_{12}$, and $F_{22}$. Then, the SDF value of the point P can be obtained via Equation (3):

(3) $$SDF(P)=\frac{1}{\left(x_{2}-x_{1})(y_{2}-y_{1}\right)}[\begin{array}{cc} x_{2}-x & x-x_{1} \end{array}]\left[\begin{array}{cc} F_{11} & F_{12}\\ F_{21} & F_{22} \end{array}\right]\left[\begin{array}{c} y_{2}-y\\ y-y_{1} \end{array}\right]$$

Based on the continuous representation of the SDF, the robot’s size can be further considered to obtain a safe and feasible region. The robot is simplified as a disk with a radius δ, and a hierarchical safety margin strategy is adopted. Specifically, during the sampling phase of PRM, the safety margin is set to δ. In the ant exploration phase, the safety margin is adjusted to 2δ to provide sufficient geometric space for subsequent trajectory optimization. Finally, during the trajectory optimization phase, the safety margin is restored to δ.

During each phase of the algorithm, the safety and feasibility of the path segments must be determined. For a continuous path segment $\bar{PQ}$, its feasibility is defined by Equation (4):

(4) $$\forall t\in [0,1],SDF\left((1-t)\cdot P+t\cdot Q\right)\geq \delta$$

in which (1−t)·P+t·Q represents the point corresponding to parameter t on the line segment. When t varies from 0 to 1, the entire line segment can be traversed.

## 3. Design of the TPP-CSACO

### 3.1. Construction of Topology and Progress Potential

#### 3.1.1. Construction, Simplification, and Connectivity Enhancement of PRM

The nodes generated by RRT* are concentrated near the path from the start to the target point, making it difficult to characterize the topology of regions far from this path. Consequently, alternative path information may be lost in complex multi-corridor environments. In contrast, the PRM’s sampling strategy distributes nodes throughout the free space rather than clustering them around the path. This allows the constructed topological graph to present more feasible paths and capture the complete connectivity structure of the environment. A robust and information-explicit PRM topological graph is the key to constructing the potential for progress. Therefore, systematic enhancements have been implemented for the sampling strategy, edge connection mechanism, and graph structure of the traditional PRM algorithm.

In this paper, a hybrid sampling strategy combining four types of sampling methods is adopted: uniform sampling, obstacle-boundary-biased sampling, Gaussian sampling, and bridge test sampling. Let $N_{sam}$ denote the total number of sampling nodes; the number of samples for each type is allocated according to the integer proportion $\lambda =(\lambda_{U},\lambda_{B},\lambda_{G},\lambda_{R})$, as given in Equation (5):

(5) $$n_{k}=\left[N_{sam}\cdot \frac{\lambda_{k}}{\sum_{i=\{U,B,G,R\}}\lambda_{i}}\right],k\in \{U,B,G,R\}$$

in which $n_{U}$, $n_{B}$, $n_{G}$, and $n_{R}$ represent the number of uniform sampling nodes, boundary sampling nodes, Gaussian sampling nodes, and bridge test sampling nodes, respectively. Subsequent sampling in this paper is performed with a ratio of 2:2:1:1. Meanwhile, a sampling refill mechanism is set up: if sampling point failure occurs for a particular sampling type, the insufficient number of samples will be supplemented by uniform sampling, ensuring the sampling density in the feasible region of the initial sampling.

After node sampling, the node set $V_{prel}=\left\{P_{i}\in R^{2}|i=1,2,\ldots ,N\right\}$ is obtained. Edge connection is then performed: for each node $P_{u}$, its K-nearest neighbors are first queried to form a candidate edge $e=(P_{u},P_{v})$, and then, edge extension equidistant sampling detection is executed on the candidate edge. Let $K_{seg}$ denote the number of sampling points along the edge; the calculation formula of the sampling points is given in Equation (6):

(6) $$\left\{\begin{array}{c} q_{k}=P_{u}+t_{k}\cdot (P_{v}-P_{u})\\ \begin{array}{cc} t_{k}=k/K_{seg}, & k=0,1,\ldots ,K_{seg} \end{array} \end{array}\right.$$

A candidate edge e is deemed feasible if and only if $\forall q_{k}\in e:SDF(q_{k})>0$. To ensure the local connectivity of the graph, a minimum constraint ${deg}_{min}=5$ is introduced. If the feasible neighbor count $P_{u}$ of the node $P_{u}$ is less than ${deg}_{min}$, the search radius is increased incrementally until $deg(P_{u})\geq {deg}_{min}$.

To address the isotropic defect of traditional KNN edge connection, an oriented expansion strategy is introduced. The 2π angular range is divided equally into eight sectors. For each node $P_{i}$, the candidate node with the closest projection distance is selected in each sector s, and the selection rule is given in Equation (7):

(7) $$P^{s}={\mathrm{argmin}}_{P_{j}\in V_{\mathrm{prel}},\theta_{j}\in \vartheta_{s}}\left((P_{j}-P_{i})\cdot u_{s}\right)$$

in which $u_{s}$ denotes the unit vector in the central direction of sector s and $\vartheta_{s}$ represents the angular range of sector s. When the edge $\bar{P_{i}P^{s}}$ is feasible, it is added to the edge set E to ensure that the node is connected by short edges in all principal directions.

The above strategy can construct the topological structure of the feasible region, but the large number of nodes and edges will increase the subsequent computational cost. Therefore, on the premise of ensuring the reachability between the start point and the end point, we simplify the graph according to the following steps to strengthen the backbone structure and retain the narrow channel characteristics:

(1) Divide the workspace into grid cells $l_{c}=0.5$ with an edge length of $\left\{C_{j}\right\}$. The start point $i_{S}$ and end point $i_{G}$ are retained compulsorily. For each cell, only the node with the maximum SDF value is retained as the representative point $P_{j}^{*}$, while the remaining nodes in the cell are removed. Subsequently, the adjacency relationships are reconstructed on the set of representative points, and infeasible edges are filtered out.

(2) For a node $P_{u}$ satisfying $\deg(P_{u})=2$ (connected only to $P_{v}$ and $P_{w}$), if the included angle of the segment formed by $P_{v}$ and $P_{w}$ is smaller than 25° and the edge $\left(P_{v},P_{w}\right)$ passes the feasibility check, then $\left(P_{v},P_{w}\right)$ is used to replace the edges $\left(P_{v},P_{u}\right)$ and $\left(P_{u},P_{w}\right)$, while $P_{u}$ is removed. This operation is executed iteratively until no nodes can be contracted.

(3) After each stage, depth-first search is used to compute the connected component function $comp:V\to \left\{1,2,\ldots ,K\right\}$, so as to check the connectivity between the start point $i_{S}$ and the end point $i_{G}$. If $comp(i_{S})=comp(i_{G})$, the simplified result is accepted; if disconnection occurs, the graph structure is rolled back to the previous stage step by step to ensure the start and end points remain connected at all times.

(4) If the start and end points are still disconnected after the rollback in the previous stage, the target component $V_{S}$ (the component containing the start point) and all components $V_{\neg S}$ that do not contain the start point are marked. A monotonically increasing search radius set $R=\left\{r_{1}<r_{1}<\cdots <r_{T}\right\}$ is defined. For each r∈R, node pairs where the distance between $P_{u}\in V_{S}$ and $P_{v}\in V_{\neg S}$ is less than r are selected to construct the candidate pair set P(r). P(r) is traversed in ascending order of distance, the feasibility of candidate edges is verified, and the connected components are updated until the connectivity between the start and end points is restored.

This subsection presents the complete construction process of the PRM topological graph, which maintains good reachability, expressiveness, and numerical stability in complex environments. It provides a high-quality topological foundation for subsequent operations.

#### 3.1.2. Topological Progress Potential

Based on the simplified PRM topological graph, a progress potential is constructed on it to provide smooth guidance information between the start and end points. The PRM topological graph is abstracted as an undirected graph $G=\left(V,E\right)$, where the node set is $V=\left\{P_{i}\in R^{2}|i=1,2,\ldots ,N\right\}$ and the edge set is $E=\left\{e=(u,v)|u,v\in V\right\}$. When constructing the progress potential, the potential of the start point $i_{s}\in V$ is set as $\varphi (i_{s})=1$ and that of the end point $i_{G}\in V$ is set as $\varphi (i_{G})=0$. The potentials of the remaining nodes satisfy the discrete harmonic equation, so as to ensure the smoothness and monotonicity of the potential.

To balance the geometric length of edges and the characteristics of the surrounding environment, for any edge $e=\left(u,v\right)$, its geometric length $L_{e}$ is taken, and the geometric length weight $W_{L}(e)$ is defined as given in Equation (8):

(8) $$W_{L}(e)=1/(e^{-6}+L_{e})$$

Meanwhile, the minimum obstacle distance $c_{min}$ of the edge is estimated using the SDF and bilinear interpolation, and the environmental compensation modulation factor $W_{c}(e)$ is given in Equation (9):

(9) $$W_{c}(e)=\left\{\begin{array}{cc} 1+\min\left(\max\left(\frac{c_{ref}-c_{min}(e)}{c_{ref}},0\right)1\right), & if\;c_{min}\left(e\right)<c_{ref}\\ 1, & otherwise \end{array}\right.$$

in which $c_{ref}$ is the clearance reference value (matching the size of the safety margin). By combining the geometric length weight and the environmental compensation modulation factor, the edge weight $W_{Lc}(e)$ is given in Equation (10):

(10) $$W_{Lc}(e)=W_{L}(e)\cdot W_{c}(e)$$

Based on the edge weights, the graph Laplacian matrix is constructed: first, the weighted adjacency matrix $A_{ij}$ is obtained; then, the degree matrix $D_{ii}$, which represents the sum of weights of all edges connecting a node to other nodes, is derived; subsequently, the weighted Laplacian matrix L is obtained. Its formulas are given in Equations (11)–(13):

(11) $$A_{ij}=\left\{\begin{array}{cc} W_{Lc}(e), & if\;e=(i,j)\in E\\ 0, & otherwise \end{array}\right.$$

(12) $$D_{ii}=\sum_{j=1}^{N}A_{ij}$$

(13) L=D−A

Nodes are reordered into boundary nodes $B=\left\{i_{S},i_{G}\right\}$ and internal nodes I, and the potential function vector ϕ is correspondingly partitioned into $\phi =\left(\phi_{I},\phi_{B}\right)$ (where $\phi_{I}$ denotes the unknown potential values of internal nodes and $\phi_{B}$ denotes the known potential values of boundary nodes). The weighted Laplacian matrix can be block-partitioned, as given in Equation (14):

(14) $$L=\left[\begin{array}{cc} L_{II} & L_{IB}\\ L_{BI} & L_{BB} \end{array}\right]$$

in which $L_{II}$ is the Laplacian submatrix corresponding to internal nodes and $L_{IB}$ is the incidence submatrix between internal nodes and boundary nodes. Based on the discrete harmonicity constraint, the core linear equation system is given in Equation (15):

(15) $$L_{II}\phi_{I}=-L_{IB}\phi_{B}$$

Within the subgraph containing $i_{S}$ and $i_{G}$, $L_{II}$ is symmetric and positive definite. Thus, the above system of equations has a unique solution, which satisfies the weighted average property and the discrete maximum-minimum principle. For any internal node u∈I, its potential value equals the weighted average of the potentials of its neighboring nodes, as given in Equation (16):

(16) $$\phi (u)=\frac{1}{\sum_{v\in N(u)}W_{Lc}(e_{uv})}\sum_{v}W_{Lc}(e_{uv})\phi (v)$$

in which N(u) denotes the neighbor set of node u. Based on this, the potential progress values of all nodes in the map can be obtained. This design provides a globally monotonic progress scale consistent with the topology, facilitating the formation of reliable global guidance in complex scenarios.

**Figure 2:**
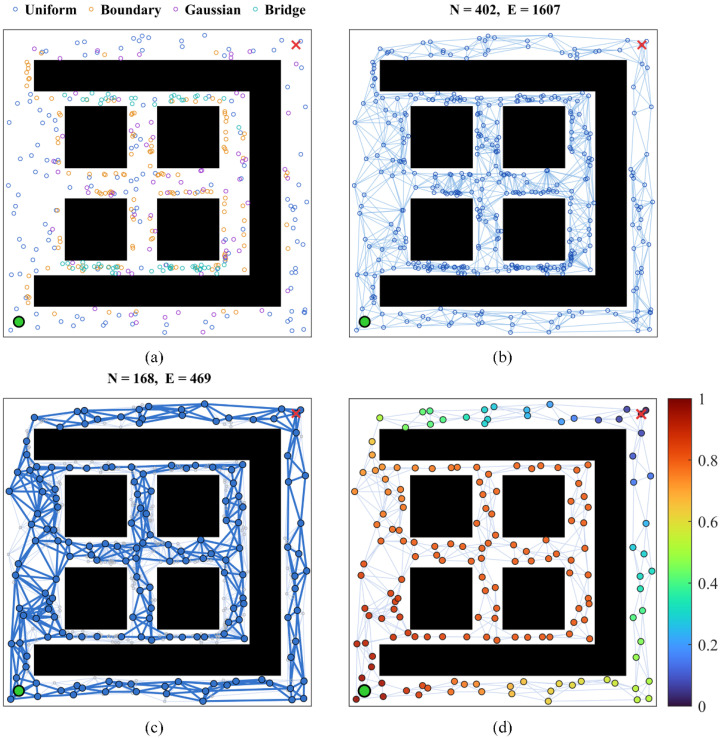
Construction of the simplified PRM and topological progress potential. (**a**) Sampling on the map. (**b**) Node connection. (**c**) Node simplification. (**d**) Topological progress potential.

As illustrated in Figure 2, the construction of the simplified PRM and the topological progress potential on the operating map is depicted.

### 3.2. Sector Partition and Decision-Making Mechanism

#### 3.2.1. Sector Partition of the Perception Circle and Internal Equal-Area Discretization

TPP-CSACO needs to select directions from the angular space. A dual-layer direction evaluation model based on sector partition and equal-area discretization is introduced as the geometric foundation for TPP-CSACO decision-making: the first layer involves sector partition for the ant’s moving directions; the second layer uses polar coordinate equal-area partition to balance the computation load and area-weighted averaging.

First, the number of moving directions to be refined (i.e., the number of sectors to be discretized, $N_{\phi }$) is determined. The central angle of each sector is denoted as $\phi_{j}$ ($j\in 1,\ldots ,N_{\phi }$), and each $\phi_{j}$ is obtained by uniformly shifting the polar angle from the current position x to the target point $x_{goal}$. The central angles of adjacent sectors are separated by a fixed angular distance. A proportional coefficient η is introduced to dynamically adjust the angular domain of the sectors, and its expression is given in Equation (17):

(17) $$\left\{\begin{array}{c} \Delta \phi =2\pi /N_{\phi }\\ \theta_{j}=\left[\phi_{j}-\eta \cdot \pi /N_{\phi },\phi_{j}+\eta \cdot \pi /N_{\phi }\right] \end{array}\right.$$

in which Δϕ represents the fixed angular distance between two adjacent central angles. When η<1, a moderate angular gap exists between sectors; when η=1, sectors are connected end to end without overlap or gaps; when η>1, sectors partially overlap, and each individual sector covers a larger area. In this paper, η=1 is adopted by default to fully cover the 2π angular range without overlap. For each sector within the interval bounded by its perception circle radius R, it is divided into $N_{r}$ equal-area rings. The equal-area constraint is applied to ensure that the area of each ring is equal, and the division formula is given in Equation (18):

(18) $$\left\{\begin{array}{c} r_{i}=R\sqrt{i/N_{r}}\\ A_{i}^{r}=\pi \cdot R^{2}/N_{r} \end{array}\right.$$

in which $r_{i}$ denotes the i ring radius and $A_{i}^{r}$ denotes the area of the i ring. The angular domain $\theta_{j}$ of each sector is evenly divided into $N_{\theta }$ columns. Let $\omega_{j}$ represent the angular width of sector j; then, the step length and the central angle of the k column can be expressed as given in Equation (19):

(19) $$\left\{\begin{array}{c} \Delta \theta =\omega_{j}/N_{\theta }=2\cdot \eta \cdot \pi /N_{\phi }\cdot N_{\theta }\\ \theta_{j,k}=\phi_{j}-\left(\eta \cdot \pi /N_{\phi }\right)+(k-0.5)\Delta \theta \\ P_{j,i,k}=x+{\bar{r}}_{i}\cdot \left[\begin{array}{c} \cos\theta_{j,k}\\ \sin\theta_{j,k} \end{array}\right] \end{array}\right.$$

in which Δθ denotes the angular step length, $\theta_{j,k}$ is the central angle of the k column, $P_{j,i,k}$ represents the central coordinate of the small area unit located in the i ring and k column of the j sector, and $\;{\bar{r}}_{i}=(r_{i-1}+r_{i})/2$ is the radial center of the i ring. The SDF(p) is used to determine the safe region attribution of each subunit in the sector, and the index of the first blocked unit in each direction is defined as given in Equation (20):

(20) $$allow(j,i,k)=\left\{\begin{array}{cc} 1, & if\;SDF(P_{j,i,k})>2\delta \\ 0, & otherwise \end{array}\right.$$

For the k column of sector j, the first blocked ring is defined as the first radially blocked ring, and its formula is given in Equation (21):

(21) f(j,k)=min{i:allow(j,i,k)=0}

When the k column is fully feasible, $f(j,k)=N_{r}+1$, and the effective passable radius is $R_{j,k}=r_{f(j,k)-1}$. The set of subunits within sector j is defined as:

(22) $$M_{j}=\left\{(i,k)|allow(j,i,k)=1\right\}$$

In the subsequent pre-screening and detailed scoring process, multiple quantities can be sampled synchronously at the center points of subunits. To aggregate the unit information into sector-level evaluations, a cosine-based angular weighting strategy is adopted, assigning higher weights to the central column. The weight formula is given in Equation (23):

(23) $$\omega_{k}=\cos\left(\pi \cdot \left|k-k_{c}\right|/3\cdot k_{max}\right)$$

in which $k_{c}=(N_{\theta }+1)/2\;$ serves as the central column index and $k_{max}=N_{\theta }/2\;$ denotes the maximum distance. The weighted effective radius of sector j is given in Equation (24):

(24) $${\bar{R}}_{j}=\left(\sum_{k=1}^{N_{\theta }}\omega_{k}\cdot R_{j,k}\right)/\sum_{k=1}^{N_{\theta }}\omega_{k}$$

By constructing a polar coordinate grid within each sector, subsequent continuous integration can be converted into a local summation over each sector. The equal-area partition method can avoid systematic area-weighting biases in the average calculation of subsequent candidate sampling points, significantly reducing the subsequent computational load.

**Figure 3:**
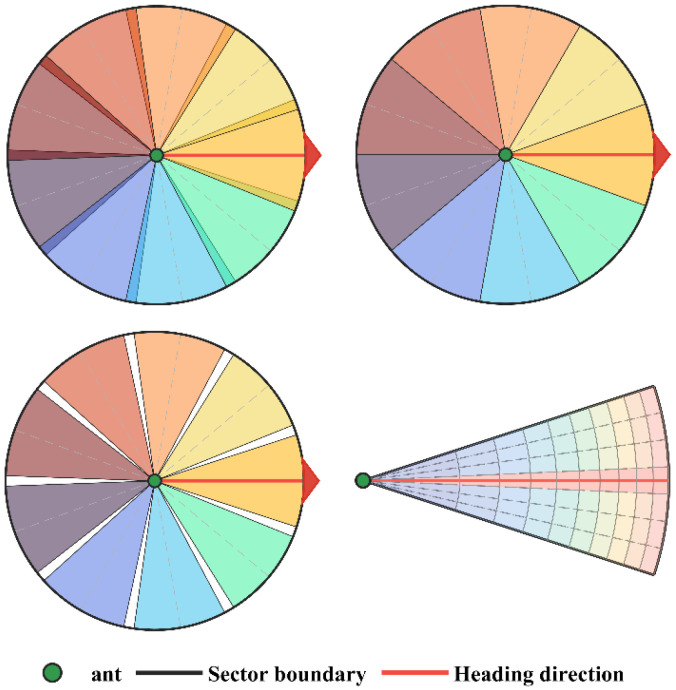
Sector division and division of internal subunits.

Regarding the computational complexity, the sector division and the division of its internal subunits are illustrated in Figure 3. Specifically, the sector division scenario for η=1 is presented in the top-left quadrant; the sector division scenario where η<1 is depicted in the top-right quadrant; the sector corresponding to η>1 is shown in the bottom-left quadrant; the internal division scenario of a single sector is displayed in the bottom-right quadrant.

#### 3.2.2. Sector Pre-Screening

Conducting a complete evaluation for all $N_{\phi }$ directions at each ant step would lead to significant computational overhead. Therefore, it is necessary to eliminate poor directions using relatively lightweight information, retaining optimal directions for subsequent detailed scoring. The pre-screening evaluation formula is defined in Equation (25):

(25) $$H_{pre}(j)=B(j)\cdot G_{geo}(j)\cdot G_{\tau }(j)$$

in which B(j) is the basic direction score of the j  sector, $G_{geo}(j)$ is the feasible region factor of the j sector, and $G_{\tau }(j)$ is the pheromone factor of the j sector. To calculate the basic direction score, the progress potential statistical measure over the sector units must be obtained first, as given in Equation (26):

(26) $$\left\{\begin{array}{c} S_{\phi }(j)=\frac{1}{\left|M_{j}\right|}\sum_{P\in M_{j}}\max(0,\varphi (pos)-\varphi (P))\\ {\tilde{S}}_{\phi }(j)=\min(1,\max(\frac{S_{\phi }(j)-\min(S_{\phi }(j))}{\max(S_{\phi }(j))-\min(S_{\phi }(j))},0)) \end{array}\right.$$

in which $S_{\phi }(j)$ is the average value of progress potential decline. When the number of feasible units in the sector $M_{j}=0$, $S_{\phi }(j)=0$. $\;{\tilde{S}}_{\phi }(j)$ is the normalized progress potential decline index. Then, the alignment degree between the sector’s central direction and the target direction is calculated, and its formula is given in Equation (27):

(27) $$g(j)=\max(0,\cos(\phi_{j}-\phi_{goal}))$$

in which $\phi_{goal}\;$ is the direction angle from the current position to the target. This term can increase the score of sectors oriented toward the target and suppress those oriented away from it. The final basic direction score B(j) adopts a dynamic weight fusion strategy, combining ${\tilde{S}}_{\phi }(j)$ and g(j). When the potential information is reliable, its weight is increased; otherwise, greater reliance is placed on the target direction. The formula for this is given in Equation (28):

(28) $$\left\{\begin{array}{c} B(j)=\omega_{\varphi }(j)\cdot {\tilde{S}}_{\phi }(j{)}^{k}+(1-\omega_{\varphi }(j))\times g(j{)}^{\beta (j)}\\ \omega_{\varphi }(j)=0.4+(4\cdot {\tilde{S}}_{\phi }(j)-0.6)/(1+\exp(-j))\\ \beta (j)=\beta_{min}+(\beta_{max}-\beta_{min})\cdot {\tilde{S}}_{\phi }(j) \end{array}\right.$$

in which k denotes the trend power exponent, $\omega_{\varphi }(j)\in [0.4,\;0.8]$ is the dynamic weight factor controlled by ${\tilde{S}}_{\phi }(j)$, and $\beta (j)\in [\beta_{min,}\beta_{max}]$ is the amplification/suppression coefficient. When a sector shows significant potential drop in the progress potential, even if its directional alignment g(j) is small, ${\tilde{S}}_{\phi }(j)$ can still yield a definite basic directional score in this direction. This helps in navigating around trap regions and avoids falling into local optima due to overreliance on g(j). For the feasible region factor $G_{geo}(j)$, it is necessary to calculate the coverage rate inside the sector, radial feasible depth, and obstacle avoidance quality. The formulas for these indicators are given in Equations (29)–(31):

(29) $${\mathrm{cov}}_{j}=\frac{1}{N_{r}\cdot N_{\theta }}\sum_{i=1}^{N_{r}}\sum_{k=1}^{N_{\theta }}allow(i,k)$$

(30) $$dep_{j}=median_{k}\left(\sqrt{\left(f(j,k)-1\right)/N_{r}}\right)$$

(31) $$qual_{j}=\min(1,\max(\frac{1}{\left|M_{j}\right|}\sum_{P\in M_{j}}SDF(P_{j,i,k}^{pre}),0))$$

in which ${cov}_{j}\;$ denotes the coverage rate, $dep_{j}$ is the radial feasible depth, and $qual_{j}$ represents the obstacle avoidance quality. By taking the geometric mean of these three indicators and mapping the result to the interval [0.2,1], the feasible region factor $G_{geo}(j)$ can be obtained, with its formula given in Equation (32):

(32) $$\left\{\begin{array}{c} g_{geo}=({\mathrm{cov}}_{j}\cdot dep_{j}\cdot qual_{j}{)}^{1/3}\\ G_{geo}(j)=0.2+0.8\times \min(1,\max(g_{geo},0)) \end{array}\right.$$

This design can retain a base score of 0.2, avoiding the complete occlusion of potential feasible directions. For the pheromone factor $G_{\tau }(j)$, a pheromone trigger mechanism is adopted: first, pheromone sampling is performed on the sector direction to obtain the directional pheromone $\tau_{pre}(j)$; then, gain triggering is determined using the initial pheromone $\tau_{0}$ and the trigger threshold coefficient $\mu_{pre}$, with the formulas given in Equations (33) and (34):

(33) $$z_{pre}(j)=\left\{\begin{array}{cc} 1, & \tau_{pre}(j)\geq \tau_{0}\cdot \mu_{pre}\\ 0, & otherwise \end{array}\right.$$

(34) $$G_{\tau }(j)=1+z_{pre}(j)$$

After obtaining the rough scores of each sector via the pre-screening evaluation formula and sorting them, the number of sectors to be retained is determined in accordance with Equation (34):

(35) $$K_{pre}=\max\left(K_{min},\left[\eta_{pre}\cdot N_{\phi }\right]\right)$$

in which $K_{min}$ denotes the minimum number of sectors to retain and $\eta_{pre}=0.55$ is the retention ratio. By truncating the sorted sectors, the set of sectors that proceed to subsequent evaluation, denoted as $K=\{j_{1},\ldots ,j_{K_{pre}}\}$, is obtained.

The pre-screening uses a sector-based retention strategy, where 55% of candidate directions are retained by default, with a specified minimum retention quantity. Such a lenient screening criterion provides sufficient error tolerance margin for deviations in the scoring process. The pre-screening score integrates two complementary indicators, namely the global progress potential decline index and the target alignment degree, which reflect the global accessibility of the direction and the geometric relationship between the direction and the end point, respectively. Even if a single indicator suffers from local computational errors, the other indicator can still ensure a high overall score for the direction, thereby retaining high-quality directions in the candidate set. The lower bound of the geometric term is set to 0.2, which ensures that no direction will be eliminated due to obstacle occlusion. High-quality directions can still be included in the candidate set by virtue of the advantages of other terms. The base value of the pheromone term is set to 1, which imposes no negative impact on directions without prior experience but does have a high score in other terms.

#### 3.2.3. Detailed Scoring Model

After pre-screening, the candidate sector set is reduced from $N_{\phi }$ to $K_{pre}$. Compared with the low-resolution fast screening of pre-screening, the detailed scoring has two distinct features: it uses denser subunit to sample the potential field, improving the accuracy of environmental information; it introduces a boundary fine interpolation mechanism to precisely locate the safe passage boundary, providing a geometric basis for elastic step-length constraints.

**Figure 4:**
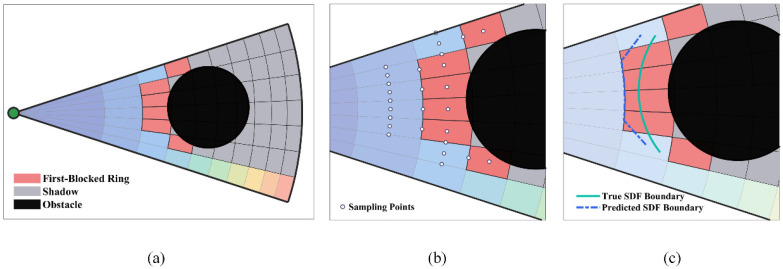
Process for obtaining equipotential lines. (**a**) First blocked ring. (**b**) Five-point sampling. (**c**) Prediction of equipotential lines.

The first blocked ring index f(j,k) defined by Equation (20) can only achieve ring-level blocking judgment and cannot precisely locate the radial position of the safe boundary (the 2δ equipotential line). Therefore, a five-point boundary sampling mechanism is introduced. For the first blocked ring $f_{b}=f(j,k)$, additional SDF sampling is performed at the following five key positions, as specified in Equation (36):

(36) $$\left\{\begin{array}{c} d_{safe}=SDF(pos+{\bar{r}}_{f_{b}-1}\cdot e_{j,k})\\ d_{entry}=SDF(pos+r_{f_{b}-1}\cdot e_{j,k})\\ d_{block}=SDF(pos+{\bar{r}}_{f_{b}}\cdot e_{j,k})\\ d_{left}=SDF(pos+{\bar{r}}_{f_{b}-1}\cdot e_{j,k-0.5})\\ d_{right}=SDF(pos+{\bar{r}}_{f_{b}-1}\cdot e_{j,k+0.5}) \end{array}\right.$$

in which $e_{j,k}={\left[\cos\theta_{j,k},\sin\theta_{j,k}\right]}^{T}$ is the direction unit vector and $e_{j,k\pm 0.5}$ denotes the direction of the angular column boundary. The safe ring center ${\bar{r}}_{f_{b}-1}$ is located in the unblocked area; the entrance of the first blocked ring $r_{f_{b}-1}$ (i.e., the outer boundary of the safe ring) is the boundary between the safe and dangerous areas; the center of the first blocked ring ${\bar{r}}_{f_{b}}$ is located inside the blocked area. The geometric relationship is illustrated in Figure 4.

Assume that the SDF varies approximately linearly in the radial direction. The radial position of the 2δ equipotential line is estimated via linear interpolation. The processing is divided into two cases based on the safety state of the entrance point: when the entrance is in a dangerous state ($d_{entry}<2\delta$), the $d_{entry}<2\delta$ equipotential line lies between the safe ring center and the entrance, and the interpolation formula is given in Equation (37):

(37) $$\left\{\begin{array}{c} t_{1}=(d_{safe}-2\delta )/(d_{safe}-d_{entry})\\ r_{\mathrm{int}erp}(j,k)={\bar{r}}_{f_{b}-1}+t_{1}\cdot (r_{f_{b}}-{\bar{r}}_{f_{b}-1}) \end{array}\right.$$

When the entrance is in a safe state ($d_{entry}\geq 2\delta$), the 2δ equipotential line lies between the entrance and the center of the first blocked ring, and the interpolation formula is given in Equation (38):

(38) $$\left\{\begin{array}{c} t_{1}=(d_{entry}-2\delta )/(d_{entry}-d_{block})\\ r_{\mathrm{int}erp}(j,k)=r_{f_{b}-1}+t_{2}\cdot ({\bar{r}}_{f_{b}}-r_{f_{b}-1}) \end{array}\right.$$

The above radial interpolation can reasonably predict the radial position of the equipotential line, but it cannot eliminate the interference of lateral obstacles. When $\min(d_{left},d_{right})<2\delta$, the interpolation result is contracted to the safety ring center for lateral obstacle intrusion, with the formula given in Equation (39):

(39) $$r_{\mathrm{int}erp}(j,k)=\min(r_{\mathrm{int}terp}(j,k),{\bar{r}}_{f_{b}-1})$$

To enhance conservatism, the minimum value of three adjacent columns is adopted to avoid excessively large step estimations caused by single-column errors, as given in Equation (40):

(40) $$r_{final}(j,k)=\min\{r_{\mathrm{int}erp}(j,k),r_{\mathrm{int}erp}(j,k-1),r_{\mathrm{int}erp}(j,k+1)\}$$

After the above processing, the safe passage radius for each angular column in the sector is given by Equation (41):

(41) $$r_{safe}(j,k)=\max\left(0,r_{final}(j,k)\right)$$

By applying the angular-column cosine weighting from Equations (23) and (24), the weighted safe passage radius ${\bar{R}}_{j}^{safe}$ of sector j is obtained, and the normalized passage depth factor is defined as Equation (42):

(42) $$\xi_{j}={\left({\bar{R}}_{j}^{safe}/R\right)}^{\alpha_{Geo}}$$

in which $\alpha_{Geo}$ denotes the obstacle penalty intensity. This factor reflects the radial passage capability of the sector direction and is used for depth correction of the geometric term. Based on geometric analysis and multi-field quantity sampling, the detailed scoring formula for the candidate sector is shown in Equation (43):

(43) $$H_{coa}=G_{dir}(j)\cdot T_{\tau }(j)\cdot G_{geom}(j)$$

in which $G_{dir}(j)$ denotes the directional guidance factor, $T_{\tau }(j)$ represents the pheromone main term, and $G_{geom}(j)$ refers to the geometric term. These three terms are fused in a multiplicative manner, functioning independently yet collaboratively. To achieve reasonable normalization of on-site progress and avoid interference from local extreme values, a global normalization scheme based on gradient statistics of PRM edges is adopted. By taking the 98th quantile $k_{98}$ of the gradient $\nabla_{e_{\phi }}$ of the progress potential on each PRM edge as the typical maximum gradient, the global potential drop normalization scale is determined as $\Delta \varphi_{max}=k\cdot R$; this scale is based on the “maximum per-step progress potential drop value” while matching the perception circle R. The potential progress of the subunit is defined as Equation (44):

(44) $$e_{\varphi }\left(i,k\right)=\min(1,\max(\frac{\varphi \left(pos\right)-\varphi \left(i,k\right)}{\Delta \varphi_{max}},0))$$

in which φ(pos) denotes the progress potential value at the current position. The direction orientation factor $G_{dir}(j)$ measures the alignment degree between the central direction of the sector and the target direction and dynamically adjusts the response intensity in combination with potential progress. First, the average potential progress of the allowed units within the sector is calculated, with the formula given in Equation (45):

(45) $${S^{\prime }}_{\phi }(j)=\frac{1}{\left|M_{j}\right|}\sum_{(i,k)\in M_{j}}e_{\phi }(i,k)$$

Since the g(j) obtained from pre-screening after direction refinement cannot effectively distinguish adjacent sectors, the sector division model is instead used to linearly map the target contribution degree of each direction, whose expression is given in Equation (46):

(46) $$g^{\prime }(j)=\left\{\begin{array}{cc} 1-\left(\lambda_{lin}\cdot N_{\phi }\cdot \left|\phi_{j}-\phi_{goal}\right|\right)/2\pi , & if\;\left|\phi_{j}-\phi_{goal}\right|\leq \frac{\pi }{2}\\ 0, & otherwise \end{array}\right.$$

in which $\lambda_{lin}$ is the linear gain slope. By substituting ${S^{\prime }}_{\phi }(j)$ into Equation (28), the amplification coefficient β(j) is obtained, which forms the direction orientation factor together with $g^{\prime }(j)$. The formula is given in Equation (47):

(47) $$G_{dir}(j)=(1+g^{\prime }(j){)}^{\beta (j)}$$

The pheromone main term $T_{\tau }(j)$ reflects the accumulation intensity of the swarm’s historical exploration experience in the sector direction. First, the average pheromone value of the allowed units within the sector is calculated, with the formula given in Equation (48):

(48) $$\bar{\tau }(j)=\frac{1}{\left|M_{j}\right|}\sum_{(i,k)\in M_{j}}\tau (j,i,k)$$

To avoid extreme values dominating the score, a saturation normalization method is adopted to map the pheromone to a bounded interval, and the pheromone main term is obtained via exponential amplification. The formula is given in Equation (49):

(49) $$\left\{\begin{array}{c} \hat{\tau }(j)=\bar{\tau }(j)/(\bar{\tau }(j)+\tau_{sat})\\ T_{\tau }(j)=\exp\left(\omega_{\tau }\cdot \hat{\tau }(j)\right) \end{array}\right.$$

in which $\tau_{sat}$ is the saturation constant and $\omega_{\tau }$ is the pheromone exponential weight. This design ensures that the pheromone remains non-negative and provides exponential-level benefits to regions with high pheromone. The geometric term $G_{geom}(j)$ is used to comprehensively evaluate the unit-level geometric quality and radial passage depth of the sector. The geometric quality is obtained, and the unit-level geometric quality is aggregated by angular columns; the formulas are given in Equations (50) and (51):

(50) $$q(i,k)=e_{\phi }(i,k)\cdot c(i,k)$$

(51) $${\bar{q}}_{k}=\frac{1}{n_{k}}\sum_{i,k\in M_{j}}q(i,k)$$

in which $n_{k}=\left|\left\{i:(i,k)\in M_{j}\right\}\right|$. Unit-level geometric quality is aggregated into sector-level geometric direction quality $\tilde{q}(j)$ by angular columns via Equation (23). By combining the passage depth factor in Equation (42), the correction formula for the sector’s geometric direction quality is given in Equation (52):

(52) $$G_{geom}(j)=\tilde{q}(j)\cdot \xi_{j}$$

Through integration via Equation (43), the scoring set of candidate sectors is obtained as $K^{\prime }=\{(j,H_{coa}(j))|j\in K_{pre}\}$. This scoring formula incorporates three core dimensions: directional guidance, pheromone, and geometric feasibility. Its multiplicative fusion design not only embodies the guiding role of the global potential field, but also accommodates the detailed structure of local geometry and pheromone.

#### 3.2.4. State Transition Probability

In the classical ACO algorithm, the next discrete node is selected by ants based on the product of pheromone concentration and heuristic information. Since no predefined node set exists in continuous-space path planning, ants need to select the movement direction from candidate sectors. To balance convergence and exploration robustness, as well as to match the sector scoring model, the state transition probability of the traditional ACO is adjusted. A temperature-regulated softmax mechanism is introduced to achieve the balance between exploration and exploitation, and its formulas are presented in Equations (53)–(55):

(53) $$l(j)=\ln\left(\max\left(H_{coa}(j),\varepsilon \right)\right)/T$$

(54) $$l^{\prime }(j)=l(j)-\max(l(k))$$

(55) $$P\left(j\right)=\exp\left(l^{\prime }(j)\right)/\sum_{i}^{N_{\phi }}\exp\left(l^{\prime }(j)\right)$$

in which T is the temperature parameter, $\varepsilon =10^{-12}$ denotes the numerical protection constant, l(j) represents the logarithmic value converted from the original score $H_{coa}(j)$, $l^{\prime }(j)$ is the value after numerical stabilization, and $P\left(j\right)$ refers to the final state transition probability. When $T\to 0^{+}$, the differentiation of $l^{\prime }(j)$ is amplified, causing the current algorithm to be more inclined to select the direction with the highest score; when T→∞, $l^{\prime }(j)\to 0^{+}$; at this point, the score differences across all directions are minimized, and the ant is more prone to random exploration. Finally, the selected sector j and the corresponding movement direction $\phi_{j}$ are determined via the roulette wheel selection method.

The main advantages of the temperature-based logarithmic softmax state transition probability are as follows: The probability model is decoupled from the specific physical, geometric, and pheromone designs, allowing the hierarchical components to be combined in any reasonable manner while always ensuring that a higher score corresponds to a greater probability, resulting in a clear and scalable structure. Secondly, the temperature T provides a simple and controllable “exploration-exploitation” adjustment, which means it can be applied in scenarios with large score differences or significant scale changes and can balance global search and local development. Meanwhile, this scheme can also be matched with subsequent elastic step-length adjustments and realize trap breakthrough through the “stagnation-temperature rise” mechanism.

### 3.3. Ant Movement and Pheromone Update

#### 3.3.1. Elastic Step Size and Direct Connection Scheme

After the moving direction $\phi_{j}$ is determined, the actual moving step size needs to be confirmed. Due to the geometric complexity of obstacles in continuous space, a fixed step size cannot balance efficient traversal in open areas and safe navigation through narrow channels. Thus, an elastic step size mechanism is introduced, combined with the “frustration-induce temperature rise” mechanism, which enables adaptive step size adjustment and enhanced exploration. The primary constraint of the elastic step size is geometric safety, i.e., the step size does not exceed the passable distance in the current direction. The nominal step size is calculated by integrating two geometric upper bounds: the maximum expected step size is set equal to the sensing circle radius, both denoted as R; the safe passage radius $R_{safe}$ is obtained from the angular column closest to the selected direction $\phi_{j}$. The nominal step size is defined as the minimum of these two values, and the corresponding formula is given by Equation (56):

(56) $$R_{nom}=\min(R,R_{safe})$$

It can ensure that the nominal step size does not violate any geometric constraints. To enhance the breakthrough capability of the ant when it is trapped, a frustration-induced rise mechanism based on the degree of frustration is introduced. Let f(t)∈[0, 1] denote the frustration degree at the t step, whose formulas are given by Equations (57) and (58):

(57) $$f(t+1)=\lambda^{\prime }\cdot f(t)+(1-\lambda^{\prime })\cdot I_{t}$$

(58) $$\alpha_{t+1}=\left\{\begin{array}{cc} 0.5, & if\;I_{t}=1\\ 1, & otherwise \end{array}\right.$$

in which $\lambda^{\prime }$ is the frustration memory coefficient and $I_{t}$ denotes the movement flag. The movement indicator $I_{t}$ is set to 0 upon successful movement and 1 upon failure. The degree of frustration is also used to dynamically adjust the temperature parameter in the state transition probability. When f(t)>0.3, a power function mapping is employed to calculate the dynamic temperature, and the corresponding formula is given by Equation (59):

(59) $$T(f)=T_{0}+\left(T_{max}-T_{0}\right)f^{q}$$

in which $T_{0}$ serves as the temperature baseline, $T_{max}$ acts as the maximum temperature upper limit, and q denotes the temperature rise rate. When f(t)≈0, $T(f)\approx T_{0}$, with heuristic guidance being dominant. When f(t)≈1, $T(f)\approx T_{max}$; the probability differences across all feasible directions are narrowed, thereby enhancing the breakthrough capability. By integrating geometric safety and frustration scaling, the formula for calculating the actual moving step size is given by Equation (60):

(60) $$R_{use}=\max(R_{min},R_{nom},\propto_{t})$$

in which $R_{min}$ acts as the minimum step size constraint, which prevents stagnation caused by an excessively small step size. To avoid the ant from infinite looping in the unsolvable region, an obstruction counter is introduced to record the ant’s continuous obstruction status, and the corresponding formula is given by Equation (61):

(61) $$H(t+1)=\left\{\begin{array}{cc} H(t)+1, & if\;f(t+1)>f_{high}\\ 0, & otherwise \end{array}\right.$$

in which $f_{high}$ is the obstruction threshold. When the value of the obstruction function exceeds this obstruction threshold, the counter initiates counting; when H(t)=6, the ant self-destructs. When the ant approaches the target, a direct connection strategy is introduced to avoid lingering near the end point. When $\left|goal-pos\right|<R$, the formula for the SDF values of the key sampling points in the subunits of the angular column corresponding to the target direction is given by Equation (62):

(62) $$d_{center}^{\prime }=SDF(pos+{\bar{r}}_{i}\cdot e_{goal}),\;i=1,2,\ldots ,i_{goal}$$

in which $e_{goal}={\left[\cos\phi_{goal},\sin\phi_{goal}\right]}^{T}$ is the unit vector pointing to the target, ${\bar{r}}_{i}\;$ denotes the central radius of the i ring, and $i_{goal}$ refers to the radial ring index where the target is located. Along the direction from the current position to the target, if the SDF values of all central points are no less than the safety margin 2δ and SDF(goal)≥δ, the ant is allowed to move directly to the target end point.

In this section, a complete mobile execution mechanism is established: in regular scenarios, adaptive adjustment of elastic step size is implemented; in dilemma scenarios, frustration-induced rise mechanism is employed to enhance the exploration and breakthrough capability; when approaching the target, the direct connection strategy is prioritized; self-destruction is triggered upon excessive obstruction, which balances travel efficiency and robustness.

#### 3.3.2. Pheromone Update

Since the movement of ants does not occur between grid nodes, there is no grid to carry the pheromones. Compared with the Gaussian kernel, the raised-cosine kernel has both zero value and zero derivative at the boundaries, requiring no truncation processing, and the cosine function offers higher computational efficiency than the exponential function. Thus, the raised cosine kernel deposition is introduced. Consider a vertex sequence set $V=\left\{v_{0},v_{1},\ldots ,v_{m}\right\}$ for a path. The length of the k segment is $l_{k}=|v_{k+1}-v_{k}|$, and the cumulative arc length sequence is $\left\{c_{0},c_{1},\ldots ,c_{m}\right\}$ (where $c_{0}=0$, $c_{k+1}=c_{k}+l_{k}$, and $c_{m}=L$ denotes the total length of the path). The polyline segment is represented as a function γ(s) parameterized by the arc length s, which is given by Equation (63):

(63) $$\gamma (s)=v_{k}+\left[(s-c_{k})/l_{k}\right]\cdot (v_{k+1}-v_{k}),\;s\in c_{k},c_{k+1}$$

The path is resampled equidistantly with a step size of ds=0.15R, yielding the coordinates of the sampling points $\gamma (s_{i})$ and the cumulative arc lengths $s_{i}=i\cdot ds,\;i=0,1,\ldots ,N,$ and N=L/ds. The raised cosine kernel is introduced to establish the single-point deposition formula, which is given by Equation (64):

(64) $$K(r;R)=\left\{\begin{array}{cc} \left(1+\cos(\pi r/R)\right)/2, & 0\leq r\leq R\\ 0, & r>R \end{array}\right.$$

in which $T_{line}$ represents the peak path deposition intensity. The formula of the pheromone deposition model, derived via the discretization of the Riemann sum, is given as Equation (65):

(65) $$\tau_{p}(x,T_{line},R)=T_{line}\cdot (ds/R)\sum_{i=0}^{N}K(|x-\gamma (s_{i})|;R)$$

**Figure 5:**
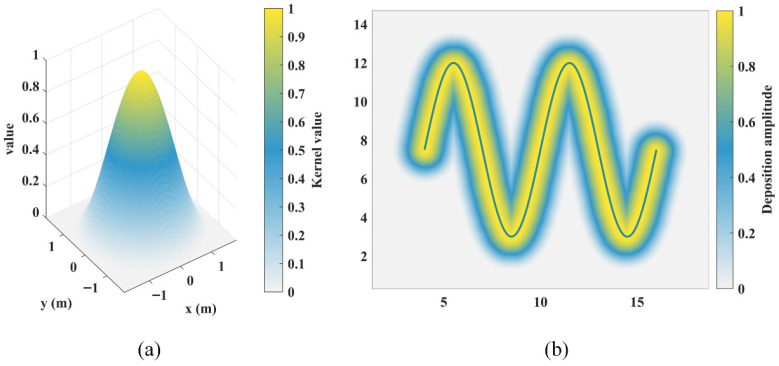
Pheromone deposition. (**a**) Single-point deposition when R=1. (**b**) Pheromone deposition along the path.

In this model, the deposition peak is controlled by adjusting $T_{line}$, while the pheromone diffusion width is regulated by tuning R. Its deposition effect is illustrated in Figure 5.

Regarding the pheromone update rule, let $U_{k}$ denote the set of points on the map that are within a distance R from the k path. Local pheromone update is executed immediately after each ant reaches the target, acting on the neighborhood of that ant’s path; its formula is given by Equation (66):

(66) $$\tau_{new}(x)=\left\{\begin{array}{cc} (1-\rho_{loc})\tau (x)+\rho_{loc}\tau_{0}, & x\in U_{k}\\ \tau (x), & otherwise \end{array}\right.$$

Global pheromone update is executed uniformly at the end of each generation. Batch deposition is performed based on the paths of all target-reaching ants, and its formulas are given by Equations (67)–(69):

(67) $$\tau_{new}(x)=(1-\rho )\tau (x)+\rho \Delta \tau (x)$$

(68) $$\Delta \tau (x)=\sum_{k=1}^{M_{ar}}\Delta \tau_{k}(x)$$

(69) $$\Delta \tau_{k}(x)=\left\{\begin{array}{cc} T_{line}=Q/L_{k}, & \mathrm{ant}\;k\;\mathrm{passes}\;\mathrm{through}\;\mathrm{path}\;p_{i}\;\mathrm{in}\;\mathrm{this}\;\mathrm{iteration}\\ 0, & otherwise \end{array}\right.$$

in which $\rho_{loc}$ denotes the local pheromone evaporation coefficient, ρ is the global pheromone evaporation coefficient, and $\tau_{0}$ represents the baseline pheromone concentration. Q is the deposition constant, $L_{k}$ stands for the geometric length of the k path, and $M_{ar}$ is the number of ants that reach the target in the current generation.

### 3.4. Trajectory Optimization

Ants that successfully reach the target generate a collision-free polygonal trajectory $V=\left\{v_{0},v_{1},\ldots ,v_{m}\right\}$. However, due to the exploration characteristic of direction refinement, the path tends to exhibit jitter, which is unfavorable for trajectory tracking and velocity planning of the robot. Therefore, continuous spatial path smoothing is performed in two stages: local low-pass smoothing and Bézier curve smoothing. Several rounds of local low-pass iterations are conducted on the inner points $v_{i}(i=1,2,\ldots ,m-1)$ of the path. In a single iteration, the formula for constructing the candidate position of the i point is given by Equation (70):

(70) $${\hat{v}}_{i}=\alpha \cdot v_{i-1}+(1-2\alpha )\cdot v_{i}+\alpha \cdot v_{i+1}$$

in which α denotes the smoothing weight. To prevent points from being overstretched, a constraint is imposed on the point displacement, and its formula is given by Equation (71):

(71) $$v_{i}^{cand}=v_{i}+\min\left(1,\frac{\delta \cdot \bar{l}}{\left|{\hat{v}}_{i}-v_{i}\right|}\right)\cdot \left({\hat{v}}_{i}-v_{i}\right)$$

in which $\bar{l}$ represents the average segment length of the path. The candidate point $v_{i}^{cand}$ must satisfy the safety constraint $SDF\left(v_{i}^{cand}\right)\geq \delta$, and no collision occurs between the candidate segments $l_{v_{i-1},v_{i}^{cand}}$ and $l_{v_{i}^{cand},v_{i+1}}$. After a limited number of iterations, the jitter in the path can be significantly eliminated, resulting in a pre-smoothed path. For the corner angles of the pre-smoothed path, quadratic Bézier corner smoothing is performed. By taking three consecutive points $A=v_{i-1}^{cand},\;B=v_{i}^{cand},\;C=v_{i+1}^{cand}$, and $l_{1}^{\prime }=B-A,l_{2}^{\prime }=C-B$ are defined. The formula for constructing the tangent points is given by Equation (72):

(72) $$\left\{\begin{array}{c} T_{1}=B-t\cdot (l_{1}^{\prime }/\left|l_{1}^{\prime }\right|)\\ T_{2}=B+t\cdot (l_{2}^{\prime }/\left|l_{2}^{\prime }\right|) \end{array}\right.$$

in which $t=t_{f}\cdot \min\left(\left|l_{1}\right|,\left|l_{2}\right|\right)$, where $t_{f}=0.33$ denotes the tangent point distance factor. By taking $T_{1}$, B, and $T_{2}$ as control points, the formula for constructing the quadratic Bézier curve is given by Equation (73):

(73) $$B(u)=(1-u{)}^{2}T_{1}+2(1-u)u\cdot B+u^{2}T_{2}$$

A chamfer is deemed valid if and only if $\forall u_{k}:SDF\left(B(u_{k})\right)\geq \delta$ and all connecting segments are collision-free. If the above condition is not satisfied, the chamfer is discarded, and the original inflection point is retained. Through the two-stage processing, path backtracking and jitter can be eliminated as much as possible while ensuring path safety, thereby making the trajectory more consistent with the dynamic constraints of the robot.

## 4. TPP-CSACO Path Planning Algorithm Process

**Figure 6:**
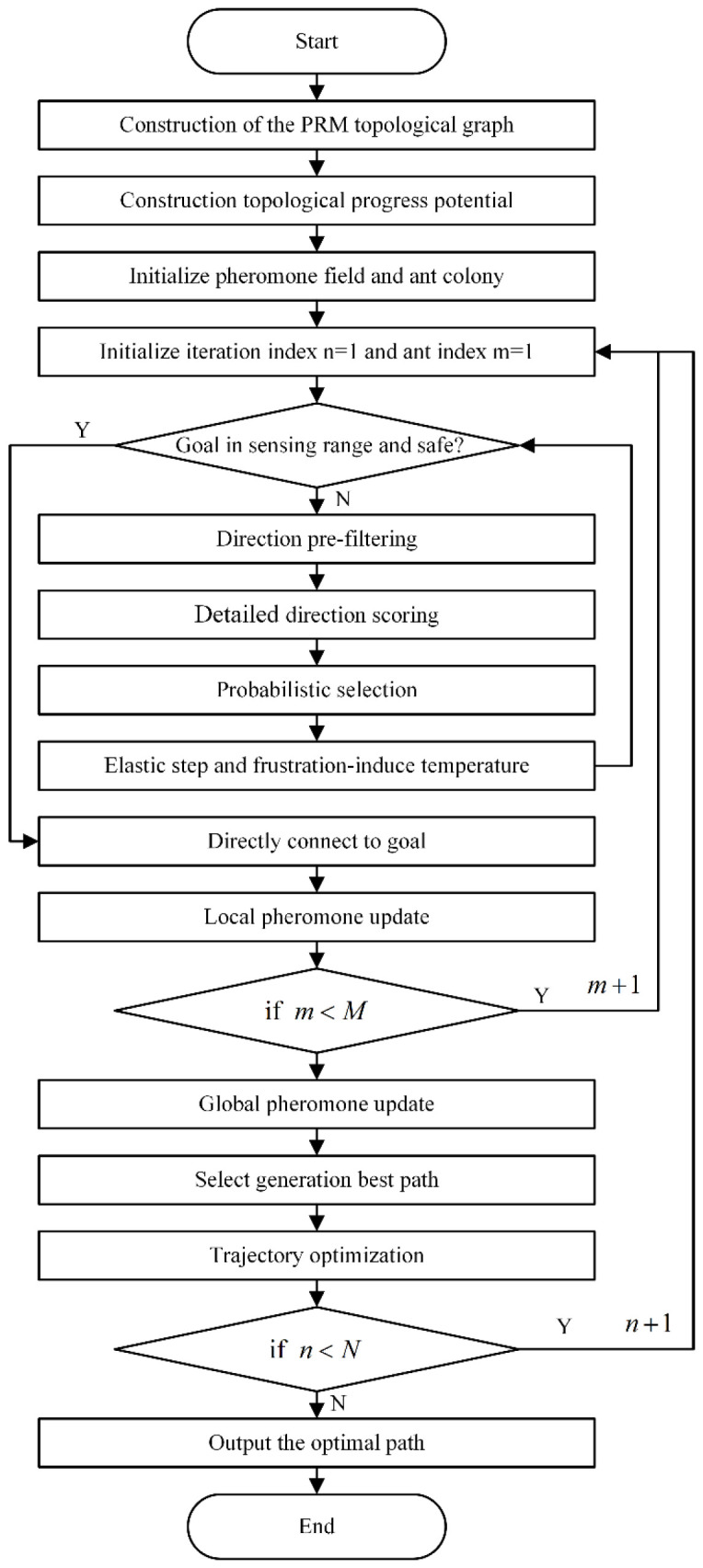
The flow diagram of TPP-ASACO.

The overall workflow of the proposed TPP-ASACO path planning algorithm is presented in this section, as illustrated in Figure 6. First, a PRM topological graph is constructed on the map, and the topological potential is calculated at its nodes to characterize the global guidance trend from the start point to the end point. Subsequently, the pheromone field is initialized on the map, and the number of ants M, iteration count n, and current ant index m are set. In the n iteration, for each ant m, the first step is to detect whether the target lies within the current sensing range. The direct path satisfies the SDF safety constraint: if the condition is satisfied, the ant is directly connected to the target, and a strip-shaped local pheromone update is performed along this path; if the target is outside the safe sensing range, pre-screening and fine scoring are conducted for the fan-shaped directions, and direction selection is carried out according to probability rules. Subsequently, the ant’s position and temperature parameters are updated based on the elastic step size and frustration mechanism, and the process returns to re-detect whether the target is visible and safe. After the current ant reaches the end point or meets the termination condition, the ant index is updated, and the above process is repeated until all M ants in this iteration are completed. After all ants in the current iteration complete the search, a global pheromone update is performed based on the pheromone regions deposited by successful paths. The optimal path of the current iteration is selected, and trajectory optimization is conducted to obtain a smooth and feasible intra-iteration optimal path. When the number of iterations reaches the upper limit N, the currently globally optimal path is output as the final planning result. The detailed pseudocode is provided in Supplementary Materials Figures S1–S4.

## 5. Simulation Experiments and Analysis

### 5.1. Algorithm Parameter Analysis

In the improved TPP-CSACO, core modules including pre-screening, detailed scoring, and elastic step size contain a large number of adjustable parameters. However, it is difficult to explain the respective action mechanisms of each parameter merely by repeatedly adjusting the parameters and running the algorithm. Moreover, coupled parameters are not conducive to system adjustment and may cause overfitting on specific maps. To address this issue, an analysis framework based on analytical models and real samples is adopted: three typical test scenarios (open area, U-shaped obstacle, and triangular obstacle) are selected. In each scenario, a sector scoring database containing information such as the forward-looking potential ${\tilde{S}}_{\phi }(j)$, direction vector  g(j), and normalized geometric depth $\xi_{j}$ is constructed in a static manner. For the positions corresponding to the parameters, the influence curves of parameter variations are plotted respectively. Other structural or weakly sensitive parameters are fixed to their default values. This approach not only enables intuitive observation of the influence pattern of the parameters but also allows for the assessment of the rationality of the default parameter configuration.

**Figure 7:**
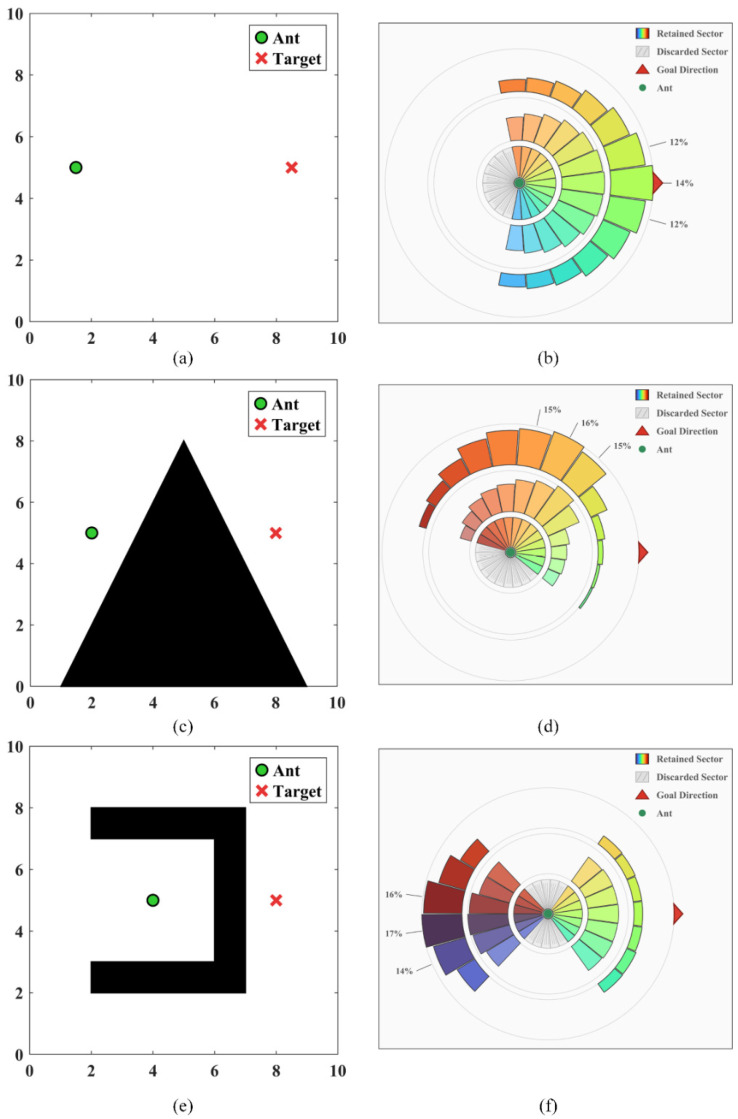
Pre-screening and scoring results of three environments under static ant conditions. (**a**) Open area environment. (**b**) Scoring results of the open area. (**c**) Triangular obstacle environment. (**d**) Scoring results of the triangular obstacle environment. (**e**) U-shaped obstacle environment. (**f**) Scoring results of the U-shaped obstacle environment.

Table 1 presents partial parameter values adopted by the current algorithm. Only in this parameter analysis is the perception radius R adjusted to 2. Figure 7 illustrates the scoring performance of three maps under the static state of ants. Among these, Figure 7b, Figure 7d and Figure 7f correspond to the reserved sector at the current position, the comparison of pre-screening scores of sectors, and the comparison of detailed evaluation scores of sectors, respectively.

**Figure 8:**
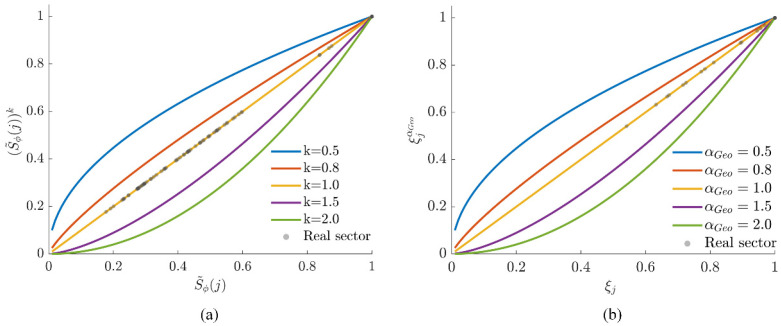
Parameter-sweep response curves for k and $\alpha_{Geo}$ (dots: real samples). (**a**) ${\left({\tilde{S}}_{\phi }(j)\right)}^{k}$ versus ${\tilde{S}}_{\phi }(j)$ under different k. (**b**) $\xi_{j}^{\alpha_{Geo}}$ versus $\xi_{j}$ under different $\alpha_{Geo}$.

Figure 8a illustrates the influence of variations in k on ${\tilde{S}}_{\phi }(j)$, where the gray scatter points represent the actual sector distributions of the three scenarios under the default value. As can be seen from Figure 8a, the actual operating points are mainly concentrated in the moderate potential drop interval rather than at the two ends of extremely optimal ${\tilde{S}}_{\phi }(j)\to 1$ or extremely poor ${\tilde{S}}_{\phi }(j)\to 0$. For k<1, the curve exhibits an upward convex shape in the moderate potential drop segment, which is equivalent to increasing the score weight of non-optimal directions and is beneficial for maintaining diversity in the pre-screening phase; for k=1, a linear influence is exhibited, and the physical gradient of the potential itself is completely retained. For k>1, the curve is obviously downward concave, which significantly reduces the scores of moderate potential drop directions and makes the algorithm more concentrated on high-quality trend directions. The term $\partial {\tilde{S}}_{\phi }(j{)}^{k}/\partial k$ indicates that the parameter sensitivity approaches 0 at both ${\tilde{S}}_{\phi }(j)\to 1$ and ${\tilde{S}}_{\phi }(j)\to 0$ and is only significant in the moderate segment. This implies that the adjustment of k mainly acts on the “sub-optimal candidate directions” with dense actual distributions, while its impact on the ranking of extremely optimal or poor directions is limited.

Figure 8b clearly demonstrates the influence of different $\alpha_{Geo}$ values on the scores of near-obstacle regions. The scatter distribution in the figure confirms that the operating points significantly affected by $\alpha_{Geo}$ are mainly those close to obstacle edges, while the impact on open regions is negligible. When $\alpha_{Geo}<1$, the score attenuation in near-obstacle regions becomes gentle. In narrow gap traversal scenarios, this setting reduces the algorithm’s repulsion against wall-hugging behavior, significantly improving the passage probability in narrow spaces. When $\alpha_{Geo}>1$, the curve drops sharply at small $\xi_{j}$ values, which means that only sectors far from the obstacle center can obtain valid scores; increasing $\alpha_{Geo}$ enables the establishment of a “harder” safety boundary around obstacles.

**Figure 9:**
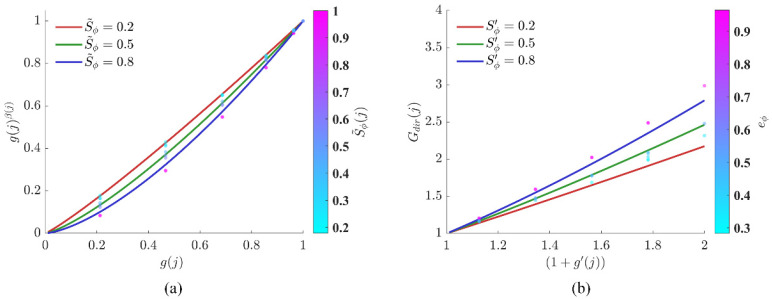
Parameter-sweep response curves of the directional terms with ($\beta_{min}$, $\beta_{max}$) fixed (dots: real sector samples). (**a**) $g(j{)}^{\beta (j)}$ versus g(j) under different ${\tilde{S}}_{\phi }$. (**b**) $G_{dir}(j)$ versus $\left(1+g^{\prime }(j)\right)$ under different $S_{\phi }^{\prime }$.

As shown in Figure 9, under the current $\beta_{min}$ and $\beta_{max}$, different potential progress indices ${\tilde{S}}_{\phi }(j)$ and average potential progress $S_{\phi }^{\prime }$ are employed to plot the curve of $g(j{)}^{\beta (j)}$ (in pre-screening) varying with the direction alignment g(j), as well as the curve of the direction pointing factor $G_{dir}(j)$ (in detailed scoring) varying with $\left(1+g^{\prime }(j)\right)$; real sector samples are overlaid on these curves. On the premise that $\beta_{min}=1$ is kept constant, a proper increase in $\beta_{max}$ will cause the curve corresponding to high-potential progress sectors to be further depressed (for pre-screening) or elevated (for detailed scoring) in the medium-to-high independent variable interval.

As illustrated in Figure 9a, when the potential drop ${\tilde{S}}_{\phi }(j)$ increases from 0.2 to 0.8, the descending amplitude of the curve is enlarged, and the curve becomes steeper in shape. Since the base number satisfies g(j)∈[0, 1], an increase in the exponent induces a monotonic increase in the function value. This implies that when the local potential drop is significant, the algorithm narrows its direction and imposes stricter nonlinear suppression on directions deviating from the target; whereas when the potential drop is weak, the curve tends to be linear, and the algorithm relaxes the tolerance to permit a wider range of trials. The scatter points in the figure indicate that the real data cover the entire dynamic adjustment range from “tolerance” to “focus.” In pre-screening, the parameter $\beta_{max}$ essentially defines the “focusing intensity” for the high potential drop region. Under the current parameters, it ensures that the algorithm converges its focus rapidly in favorable scenarios, while maintaining the necessary exploration sector in adverse scenarios—thus effectively avoiding the rigidity or blind divergence that may arise from a fixed exponent. A smaller $\beta_{max}$ makes the curve in the high-${\tilde{S}}_{\phi }(j)$ region approximate linearity, remaining relatively tolerant of deviated directions, which is beneficial for retaining more candidates in multi-objective or complex terrain; a larger $\beta_{max}$, by contrast, significantly reduces the scores of these candidates, causing the pre-screening to favor a small number of sectors that are highly aligned with the target direction.

As illustrated in Figure 9b, contrary to the inhibition logic of pre-screening, high-quality directions are enhanced through detailed scoring. As $S_{\phi }^{\prime }$ increases, the slope of the gain curve rises significantly. This implies that, under the condition of identical potential progress, nonlinear rewards are granted by the algorithm to candidate sectors approaching the target direction. The real samples in the figure confirm that the core function of this parameter is to directionally amplify high-quality candidates that conform to both the local direction and the regional trend during the final decision-making phase. In detailed scoring, the parameter $\beta_{max}$ acts as the “amplification gain” for advantageous signals. It does not interfere with the ranking of low-scoring candidates; instead, it is specifically used to widen the gap between “good” and “best” candidates, thereby significantly enhancing their relative advantages. If $\beta_{max}$ is set to a relatively low value, the final scores can be kept smoother; if $\beta_{max}$ is moderately increased, the score gap between advantageous sectors and ordinary candidates will be widened, which strengthens the decisiveness of the final decision.

A similar analysis is adopted for the parts involving pheromones, but with slight differences: instead of relying on real environmental data, the influence of pheromone intensity τ on the scoring gain $T_{\tau }(\tau )$ is directly examined through analytical formulas.

The pre-screening parameter gating $\mu_{pre}$ is essentially a threshold-based binary classifier. Instead of analyzing the influence curve of the pre-screening gating independently, it is treated as a background mechanism: the pre-screening layer only provides an eligibility assessment (to verify whether the minimum criteria are met) in the dimension of pheromone, and it does not further subdivide grades within the high τ interval where the criteria have already been satisfied.

**Figure 10:**
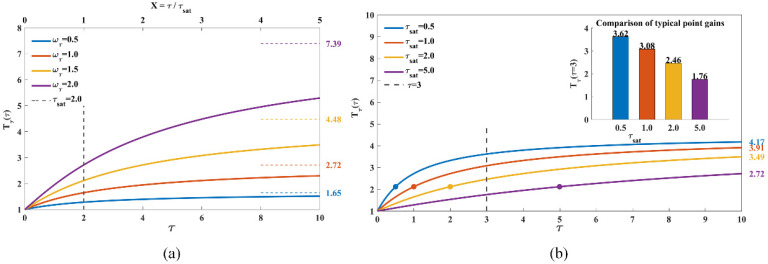
Parameter-sweep response curves of the pheromone term $T_{\tau }(\tau )$. (**a**) With $\omega_{\tau }$ fixed, $T_{\tau }(\tau )$ versus τ under different $\tau_{sat}$. (**b**) With $\tau_{sat}$ fixed, $T_{\tau }(\tau )$ versus τ under different $\omega_{\tau }$.

Figure 10a presents the influence curves of different intensity factors $\omega_{\tau }$ on the detailed scoring gain $T_{\tau }(\tau )$, under the condition that $\tau_{sat}=2.0$ (held constant). Two observation perspectives are provided by the dual horizontal axes in the figure: the lower axis corresponds to the actual pheromone concentration τ, while the upper axis represents the corresponding dimensionless intensity $X=\tau /\tau_{sat}$. From the overall morphology of the curves, $\omega_{\tau }$ determines both the gain upper limit of the pheromone term and the local discrimination capability. On one hand, each curve converges to its respective theoretical upper limit (marked by the dashed line in the figure) on the right side, which directly quantifies the maximum amplification factor achievable by mature paths. On the other hand, for the same τ or X, the larger $\omega_{\tau }$ is, the higher the slope of the curve $\partial T_{\tau }(\tau )/\partial \tau$ becomes, and the stronger the capability to resolve differences in pheromone intensity. The point where the vertical gray dashed line in the figure intersects each curve is the half-saturation point. At this location, the normalized pheromone satisfies X/(X+1)=0.5. This point divides each curve into two segments: in the left region X<1, the curve is relatively gentle with an approximately linear growth; in the right region X>1, the curve gradually flattens and tends toward saturation. In the early stage of pheromone accumulation, most sectors remain in the X<1 region. It can be observed that the curves corresponding to different $\omega_{\tau }$ values are close to each other at this stage, indicating that even if $\omega_{\tau }$ is increased, the value of $T_{\tau }(\tau )$ will only rise slowly, and the dependence of the score on $\omega_{\tau }$ is automatically suppressed. When X=0.5, $\partial \ln T_{\tau }(\tau )/\partial \omega_{\tau }\approx 0.33$, which suggests that adjusting $\omega_{\tau }$ at this stage only induces a small change in gain; when X≥2, $\partial \ln T_{\tau }(\tau )/\partial \omega_{\tau }>0.6$, so varying $\omega_{\tau }$ can significantly alter $\ln T_{\tau }(\tau )$, thereby effectively controlling the amplification factor of the pheromone term in the total score. The “converging on the left, diverging on the right” morphology reveals the mechanism of $\omega_{\tau }$: the algorithm leverages the nonlinear property of the saturation function to inherently suppress the parameter’s effect during the cold-start phase, avoiding the over-amplification of random influences in the low-pheromone-intensity region; whereas in the mature phase of the high-pheromone region, $\omega_{\tau }$ dominates the control of path utilization intensity.

Figure 10b plots the influence of $\tau_{sat}$ on $T_{\tau }(\tau )$ under the condition that $\omega_{\tau }=1.5$ is held constant. The solid circles on each curve denote the half-saturation position at $\tau =\tau_{sat}$. The black dashed line in the figure represents $\tau =\tau_{typical}=3.0$; the intersection points of this dashed line with each curve are taken as the characteristic operating points of the system in the mature stage for different $\tau_{sat}$ configurations, and the inset in the top-right corner quantifies $T_{\tau }(\tau_{typical})$ at these operating points. If the limit value of each curve as τ→∞ is regarded as “full saturation,” the positions of $\tau_{typical}$ under different $\tau_{sat}$ values can be clearly compared: When $\tau_{sat}=0.5$, $T_{\tau }(3)$ already reaches 80% of the maximum value, with the operating point lying in the latter half of the curve. Although the absolute gain is the highest, the curve has flattened significantly; subsequent increases in pheromone will only induce a negligible rise in the curve. When $\tau_{sat}=5$, $T_{\tau }(3)$ only reaches approximately 40% of the final value, leading to a relatively weak overall amplification effect. When $\tau_{sat}=1$ or $\tau_{sat}=2$, $T_{\tau }(3)$ accounts for about 55% to 70% of the maximum value: this configuration not only achieves a considerable amplification effect but also allows the curve to rise noticeably for τ>3, thus retaining a good capability to distinguish small differences in pheromone intensity.

In the elastic step size, the actual step size is strictly constrained by the geometric trust region and the half-step state machine and has been decoupled from the exploration logic. At this point, the frustration memory coefficient $\lambda^{\prime }$ and the temperature rise exponent q act as the key adjustment knobs for controlling the dynamic loop of “continuous frustration → enhanced exploration → self-termination.”

**Figure 11:**
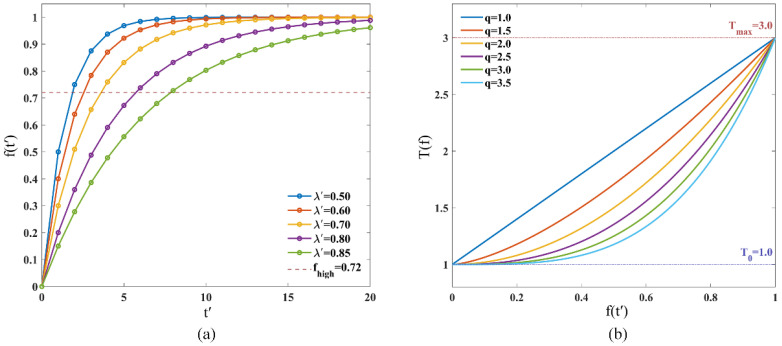
Parameter-sweep response curves of key mappings in the elastic-step mechanism. (**a**) $f(t^{\prime })$ versus the consecutive-failure steps $t^{\prime }$ under different $\lambda^{\prime }$ (with $f_{high}=0.72$ indicated). (**b**) With ($T_{0}$,$T_{max}$) fixed, T(f) versus $f(t^{\prime })$ under different q.

Figure 11a demonstrates the regulatory effect of $\lambda^{\prime }$ on the accumulation rate of the frustration degree f(t) under the limit condition of continuous frustration. The curves reveal that $\lambda^{\prime }$ essentially defines the “sensitive time window” for the ant’s congestion state: at low $\;\lambda^{\prime }$ values, merely two to three consecutive rejections suffice to drive f(t) into the high-frustration region. While this configuration offers rapid responsiveness, ants are more prone to false triggering of self-termination in narrow or crowded environments.

Figure 11b illustrates how different exponents q reshape the mapping relationship of $f(t^{\prime })\to T(f)$. When $T(f)=T_{max}$, the probability difference across all directions is reduced to one-third of its original value, which weakens directional guidance; thus, the rate and timing of temperature rise must be strictly controlled. When q>1, the temperature rise curve exhibits a distinctly concave shape. As q increases, the temperature in the low-to-moderate frustration interval is more strongly suppressed near $T_{0}$. If q=1.0 is adopted, even minor fluctuations in frustration will directly induce a temperature increase; whereas when q=2.5 is used, ants maintain T(f)<1.5 when $f(t^{\prime })<0.6$ (corresponding to the first two consecutive frustrations), preserving strong directional guidance. Only when $f(t^{\prime })$ approaches 0.9 does the temperature surge sharply to above 2.5. This “conservative in the early stage, aggressive in the later stage” response characteristic effectively balances the convergence efficiency under normal conditions and the escape capability in extreme predicaments.

$\lambda^{\prime }=0.7$ and q=2.5 constitute a set of robust parameters: they jointly establish a fault-tolerance window on the order of several consecutive rejections (about 4–5 steps before entering the high-frustration regime and around 10 steps before self-termination under continuous congestion). Within this window, the algorithm is guaranteed to possess the capability of local detouring; if continuous congestion exceeds this window, the capability to break out of predicaments is enhanced via the surging temperature.

### 5.2. Comparative Experiments and Analysis

All experiments were conducted under the same hardware and software environment to ensure the fairness of comparisons. The experimental system utilized an Intel^®^ Core i7-12700 CPU @2.0 GHz, and the experimental environment was the MATLAB 2024b platform.

To fully evaluate the performance of the algorithms, eight representative methods in the continuous-space path planning domain were selected as baselines in this section, covering RRT*, PRM, PSO, GWO, SSA, ABC, Q-learning (QL), and ACO. The TPP-CSACO algorithm proposed in this paper was designated as the core evaluation subject. The key parameters of each algorithm are listed in detail in Table 2 and Table 3. All parameters of ACO are set identically to those of TPP-CSACO, as listed in Table 4. Under the current safety margin, the minimum feasible traversal distance in the ant colony execution phase is set to four times the safety margin. The direction space is divided into 15 sectors with a central angle of 24° each, which meet the requirements for direction discrimination and prevent scoring sectors from being excessively affected by obstacles on both sides of narrow passages. For detailed scoring, 10 rings are used, with the outermost ring at 0.051R, accounting for 43% of the safety margin. This allows accurate determination of the positions of distant obstacles and improves the accuracy of direction evaluation. For the pre-screening phase, six rings are selected, with the outermost ring width set to 0.087R, which meets the detection requirements while reducing computational load.

**Figure 12:**
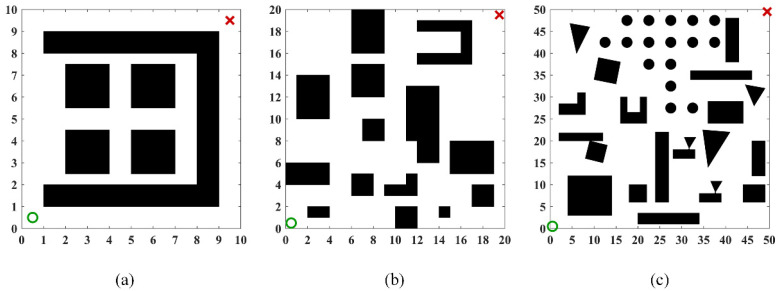
Three 2D test environments. (**a**) 10 × 10. (**b**) 20 × 20. (**c**) 50 × 50.

As shown in Figure 12, to comprehensively evaluate the path planning performance of the algorithm under varying environmental complexities, three 2D continuous-space maps with distinct characteristics were selected in this section for testing. These three maps have dimensions of 10 × 10, 20 × 20, and 50 × 50, with obstacle densities of 38.00%, 26.75%, and 23.44%, respectively, and their topological complexity increases sequentially. They correspond to the following scenarios: a locally constrained narrow-channel and deep-trap scenario, which tests the algorithm’s ability to escape local optima; a medium-density multi-obstacle discrete scenario, which evaluates the algorithm’s global optimization capability in multi-solution topologies; and a high-density multi-scale hybrid scenario, which is designed to conduct an extreme stress test on the algorithm’s safety and smoothness under large-scale, long-distance planning.

To address path quality requirements in static environments, an evaluation system consisting of four core dimensions is established: geometric efficiency, where a shorter path length L is preferable; smoothness, for which smaller values of both the total turning angle $\theta_{total}$ and the maximum turning angle $\theta_{max}$ are preferable; and safety, where the hazard zone proportion viol is defined as the percentage of the total path length occupied by hazardous segments, and this metric directly reflects the extent to which the path violates safety constraints. To conduct an unbiased, comprehensive evaluation across these dimensions, an equal-weighted composite score $J_{eq}$ is employed: the four aforementioned metrics are first normalized via the Min-Max method, and their normalized values are summed as $J_{eq}=L^{\prime }+\theta_{total}^{\prime }+\theta_{max}^{\prime }{+Viol}^{\prime }$, with a higher $J_{eq}$ indicating that the algorithm has achieved a better balance among path length, smoothness, and safety.

To eliminate the impact of randomness on algorithm performance evaluation, each algorithm is run 50 times on each map. The random seed is reinitialized for each run to ensure the independence of sampling points, initial populations, or graph construction processes. If an algorithm fails to find a collision-free path within the specified number of iterations, it is recorded as a failure. All metrics are calculated based solely on sample data from successful runs. Failed runs are excluded from metric statistics to prevent data distortion.

Table 5, Table 6 and Table 7 present detailed operational data. It can be observed that the TPP-CSACO algorithm demonstrates stable and strong performance across maps of varying complexity. In terms of path length, the TPP-CSACO algorithm is slightly inferior to the shortest-path algorithm in each scenario: compared to GWO, PSO, and SSA, its average path length increases by up to approximately 5.9% (e.g., rising from 75.708 to 80.180), while the increase in other scenarios is controlled between 0.3% and 2.9%; however, compared to the traditional ACO algorithm, the path length of TPP-CSACO can be reduced by up to approximately 50.6%. Meanwhile, the standard deviations of the path length for TPP-CSACO are 0.024, 0.219, and 1.145, respectively, which are the lowest among all algorithms in each scenario, significantly lower than the fluctuation levels of algorithms such as SSA, ABC, PRM, and ACO. In terms of path smoothness, the maximum turning angles of TPP-CSACO in the three scenarios are only 16.823°, 5.932°, and 8.720°, respectively, which are generally reduced by approximately 75% to 93% compared to the typical comparison algorithms in each scenario. In terms of safety and stability, TPP-CSACO achieves a 0% hazard zone proportion and a 100% success rate in all scenarios, while the maximum hazard zone proportion of the comparison algorithms reaches 16.54%, and the success rate of some algorithms is only 0% to 44%. In terms of comprehensive performance, TPP-CSACO ranks first among all tested environments. To verify the statistical reliability of the experimental results, the Friedman test is adopted in this paper to analyze the performance differences among various algorithms. Under three map scales, namely 10 × 10, 20 × 20, and 50 × 50, the *p*-values of all evaluation metrics are less than 0.01. Further details can be found in Supplementary Materials Table S1.

**Figure 13:**
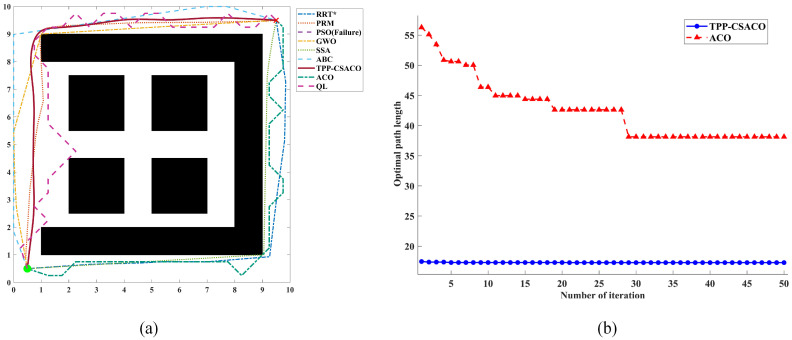
Comparison results in the 10 × 10 environment. (**a**) Final planned paths of different algorithms. (**b**) Convergence curves of the best path length versus iteration.

**Figure 14:**
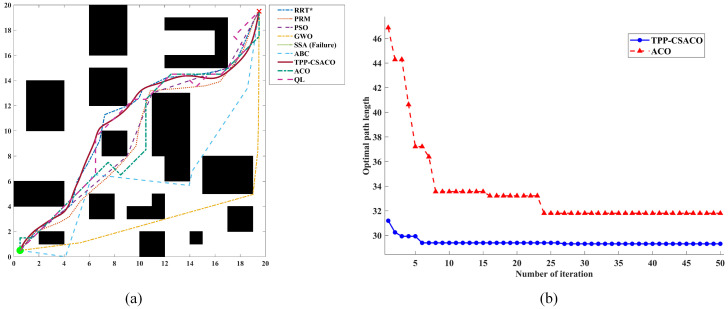
Comparison results in the 20 × 20 environment. (**a**) Final planned paths of different algorithms. (**b**) Convergence curves of the best path length versus iteration.

**Figure 15:**
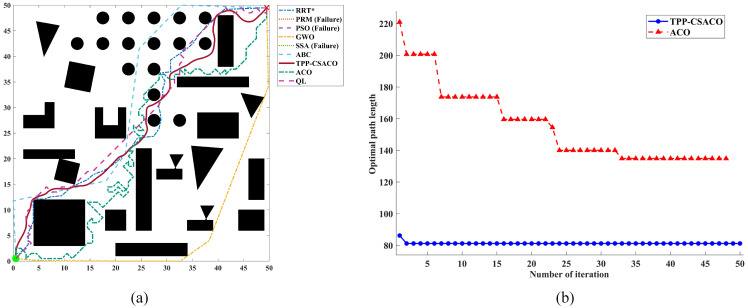
Comparison results in the 50 × 50 environment. (**a**) Final planned paths of different algorithms. (**b**) Convergence curves of the best path length versus iteration.

Figure 13, Figure 14 and Figure 15 present a set of randomly selected operation results obtained during the running process. It can be intuitively observed that, compared to the comparison algorithms, the TPP-CSACO algorithm not only maintains a relatively short path but also exhibits a smooth path morphology; meanwhile, it has a faster convergence rate than the ACO algorithm.

### 5.3. Diversity Analysis and Ablation Experiments

**Figure 16:**
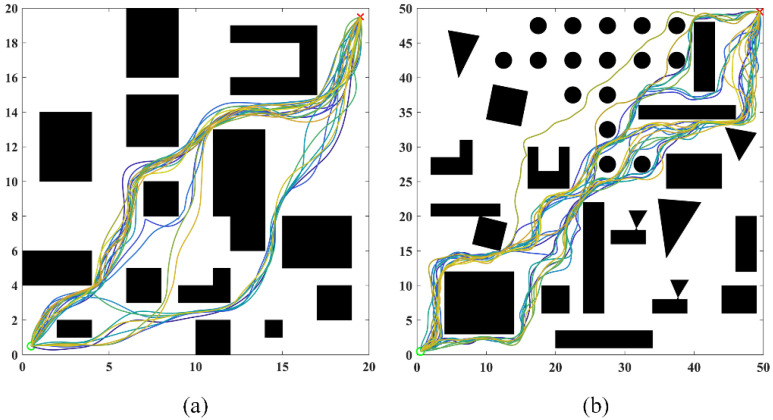
Best-path snapshots of TPP-CSACO sampled at fixed iteration intervals (single run). (**a**) 20 × 20. (**b**) 50 × 50.

The convergence rate of TPP-CSACO is notably fast across all types of environments. Based solely on the convergence curves and the final path, it is difficult to directly rule out the possibility of fast convergence to a certain locally optimal corridor. To more intuitively analyze whether TPP-CSACO maintains sufficient path diversity during the search process, a single run of TPP-CSACO is performed on the 20 × 20 and 50 × 50 environments, with parameter settings consistent with those specified earlier. The optimal path within each generation is recorded every other generation, and these paths are overlaid and plotted on the corresponding obstacle environments, as illustrated in Figure 16. By observing the distribution of the optimal paths from different generations on the environment, it can be determined that the TPP-CSACO algorithm has explored multiple corridors during the optimization process, and its exploration coverage of the feasible corridors in the environment is relatively comprehensive.

To verify the contributions of each key component in the TPP-CSACO algorithm, three ablated variants are constructed by removing, in turn, the topological progress potential, the frustration-induced temperature rise mechanism, and the elastic step length mechanism. Each variant is tested 10 times independently in a 20 × 20 environment, and three refined quantitative indicators, namely the movement rejection rate, ant arrival rate, and average number of movement steps, are recorded to distinguish the performance differences among the variants. As shown in Table 8, removing the topological progress potential results in a severe degradation in the arrival rate, with a 77% increase in the average number of steps, indicating that the global progress potential provides effective guidance during ant movement. Removing the frustration-induced temperature rise mechanism results in a 13.7% decrease in the arrival rate and a 25.7% increase in the number of steps; this mechanism facilitates ants’ escape from local traps in deep-trap regions. After eliminating the elastic step-length mechanism, the average number of steps decreases.

In contrast, the movement rejection rate increases 26.9-fold, and the ant arrival rate decreases by 13.4%, demonstrating that the elastic step length can accurately adjust ant step size across different environments. To verify the optimization performance and selection accuracy of the pre-screening strategy, 10 static verification experiments are conducted in three scenarios illustrated in Figure 7. Under the condition that the experimental settings remain unchanged across scenarios, pre-screening and detailed scoring are performed sequentially, followed by full-range detailed scoring. The running time of each stage is collected to evaluate the computational efficiency, and the accuracy of pre-screening is assessed by comparing the sector retention rates of the top-ranked results and the ranking consistency between full-range detailed scoring and pre-screened retained sectors. As shown in Table 9, the pre-screening strategy achieves a 1.3× speedup while retaining all optimal movement directions. Although some false rejections occur in complex regions, the erroneously rejected directions are all low-ranked non-optimal directions, which do not compromise the quality of the final solutions.

## 6. Conclusions

This study addresses the path planning issues existing in current ACO algorithms by proposing a topological progress potential-enhanced continuous-space ant colony path planning algorithm (TPP-CSACO), which adopts a hierarchical framework of “topological prior and continuous-space ACO.” The front end constructs a roadmap topology based on the PRM. By simplifying the PRM graph using Laplacian solution, a topological progress potential that integrates connectivity structure and clearance information is obtained, providing global search guidance. The back end operates a continuous-space ant colony under SDF constraints: it abandons the grid eight-neighborhood movement mode, employs sector division of the perception circle to refine movement directions, and adopts a pre-screening and multidimensional scoring model. By integrating the SDF safety margin, topological progress potential, and pheromone intensity distribution, a dual-field guidance mechanism that combines static prior and dynamic group experience is established. Probabilistic steering is realized through a Softmax state transition probability. By combining an elastic step size, the frustration-induced temperature rise mechanism, and the direct connection strategy, operational efficiency and the capability to break out of predicaments are balanced.

Subsequently, a simulation platform is constructed. For the key influencing parameters of the algorithm, their stable intervals are determined via static parameter formula analysis across three typical test scenarios. Subsequently, comparative verification is conducted against algorithms, including PRM, RRT*, PSO, and ACO. TPP-CSACO outperforms the other algorithms in comprehensive scoring, avoiding the excessive sacrifice of path length while ensuring no entry into hazardous areas. Compared with the traditional ACO, TPP-CSACO endows ants with stronger local perception capabilities, as well as a faster convergence rate without falling into local optima. However, TPP-CSACO does not hold an advantage in terms of computational efficiency; the single planning runtime of the current implementation is relatively long, making it difficult to compete with comparative algorithms under strict real-time constraints. This phenomenon stems primarily from two aspects: first, sector-based perception and multi-point interpolation sampling in continuous space introduce substantial constant computational overhead into single-step decision-making; second, the ant colony algorithm itself is a population-based iterative optimization approach, which requires multiple generations of iteration and pheromone updates to maintain diversity and convergence. Therefore, the current version of TPP-CSACO is more suitable as a planner for scenarios that demand high path quality (smoothness and safety margin) and topological adaptability but have relatively relaxed real-time requirements—such as offline planning or strategy generation in medium-scale scenarios. Future work will focus on further optimizing computational efficiency while ensuring path quality to enhance the competitiveness of TPP-CSACO. The sector-based perception in TPP-CSACO enables local perception, the elastic step-length strategy can adaptively regulate the movement range, and the frustration-driven temperature rise mechanism supports deadlock recovery. These mechanisms lay a solid foundation for extending the algorithm to dynamic environments. However, constrained by the real-time requirements of dynamic environments, the global progress potential requires frequent recomputation and thus fails to meet real-time response requirements. Developing local update strategies to cater to the demands of dynamic scenarios constitutes one of the future research directions.

## Figures and Tables

**Table 1:** Related parameters involved in TPP-CSACO (part one).

Parameter	$c_{ref}$	k	$\beta_{min}$	$\beta_{max}$	$\alpha_{Geo}$	$\lambda_{lin}$	δ	$\tau_{sat}$	$\omega_{\tau }$	$\lambda^{\prime }$	q	$f_{high}$	$T_{0}$	$T_{max}$	α
**Value**	0.08	1	1	1.6	1	0.22	0.06	2	1	0.7	2.5	0.72	1	3	0.25

**Table 2:** Parameters of PSO, GWO, SSA, and ABC algorithms.

Algorithm	Parameter	Value
**PSO**	Population size	100
	Number of generations	300
	Inertia weight	0.85
	Cognitive coefficient	1
	Social coefficient	1
**GWO**	Population size	50
	Number of generations	200
	α wolf weight	0.33
	β wolf weight	0.33
	δ wolf weight	0.33
**SSA**	Population size	50
	Number of generations	200
	Producer ratio	0.2
	Safety threshold	0.8
**ABC**	Population size	50
	Number of generations	200
	Abandonment limit	20

**Table 3:** Parameters of RRT*, PRM, and Q-learning algorithms.

Algorithm	Parameter	Value
RRT*	Maximum samples	20,000
	Maximum step size	0.8
	Goal sampling rate	0.1
	Goal connection radius	0.6
	Rewire radius	2.5
**PRM**	Number of samples	600
	Max neighbors (k)	15
	Connection radius	3
**Q-learning**	Learning rate (α)	0.1
	Discount factor (γ)	0.95
	Initial exploration rate ($\varepsilon_{0}$)	1.0
	Final exploration rate ($\varepsilon_{min}$)	0.01
	Exploration decay factor	0.998
	Number of training episodes	3000

**Table 4:** Related parameters involved in TPP-CSACO (part two).

Parameter	N	M	$\rho_{loc}$	ρ	Q	$\tau_{0}$	R	$N_{\phi }$	$N_{r}^{pre}$	$N_{r}$	$N_{\theta }^{pre}$	$N_{\theta }$
**Value**	50	50	0.1	0.3	1	8	1	23	6	10	5	7

**Table 5:** Results of various algorithms in the 10 × 10 map.

Algorithm	Path Length (m)			Turn (°)		Viol Percent (%)	$J_{eq}$	Success Rate
	Best	Mean	Std.	Total Turn	Max Turn			
ABC	17.441	17.990	0.345	114.457	69.414	0.398	3.290	100.0%
ACO	19.243	21.115	0.873	1468.800	126.900	0.603	1.249	100.0%
GWO	17.056	17.209	0.215	99.694	72.974	6.024	2.775	100.0%
PRM	17.100	17.241	0.075	144.424	69.951	2.548	3.249	100.0%
PSO	0.000	0.000	0.000	0.000	0.000	0.000	0.000	0.0%
RRT*	17.105	17.307	0.130	115.532	71.435	2.507	3.263	100.0%
SSA	17.038	18.056	0.912	75.566	70.505	16.535	2.454	68.0%
QL	19.837	21.165	0.632	1517.835	117.900	2.388	1.065	100.0%
TPP-CSACO	17.227	17.296	0.024	152.202	16.823	0.000	3.899	100.0%

**Table 6:** Results of various algorithms in the 20 × 20 map.

Algorithm	Path Length (m)			Turn (°)		Viol Percent (%)	$J_{eq}$	Success Rate
	Best	Mean	Std.	Total Turn	Max Turn			
ABC	29.956	32.782	1.593	249.252	90.236	0.512	2.144	100.0%
ACO	29.799	31.675	0.905	681.300	101.700	2.565	0.914	100.0%
GWO	28.324	33.948	3.416	209.678	86.628	1.029	2.078	72.0%
PRM	28.472	29.145	0.336	275.798	60.090	0.870	2.983	100.0%
PSO	28.163	28.574	0.618	139.382	53.615	4.281	2.493	44.0%
RRT*	28.650	30.061	0.835	315.494	65.236	0.867	2.679	100.0%
SSA	28.812	30.105	1.379	183.918	69.700	1.334	2.652	10.0%
QL	29.799	31.307	0.704	625.500	87.300	3.132	1.023	100.0%
TPP-CSACO	28.993	29.405	0.219	390.010	5.932	0.000	3.470	100.0%

**Table 7:** Results of various algorithms in the 50 × 50 map.

Algorithm	Path Length (m)			Turn (°)		Viol Percent (%)	$J_{eq}$	Success Rate
	Best	Mean	Std.	Total Turn	Max Turn			
ABC	79.585	89.503	4.899	305.092	98.420	0.164	3.054	82.0%
ACO	133.054	162.449	12.326	6390.000	135.000	0.381	0.805	100.0%
GWO	74.679	85.986	6.198	156.334	65.407	0.585	3.188	82.0%
PRM	86.867	92.677	6.461	1208.633	96.077	0.384	2.847	18.0%
PSO	74.213	75.708	0.731	161.084	56.051	2.190	2.768	26.0%
RRT*	76.087	82.004	3.849	742.210	70.302	1.395	2.873	100.0%
SSA	79.844	93.638	6.354	105.298	72.713	2.473	2.695	40.0%
QL	79.154	83.089	1.387	1597.500	93.600	0.976	2.542	100.0%
TPP-CSACO	78.157	80.180	1.145	1076.741	8.720	0.000	3.854	100.0%

**Table 8:** Ablation study results for key algorithm modules.

Variant	Move Rejection Rate	Ant Arrival Rate	Average Steps
TPP-CSACO	0.375%	97.417%	42.899
w/o Topological Progress Potential	2.354%	59.125%	75.911
w/o Frustration-Induced Temperature Rise	2.087%	84.083%	53.924
w/o Elastic Step Length	10.081%	84.417%	39.901

**Table 9:** Efficiency and accuracy evaluation of sector pre-screening.

Environment	Pre-Screening	Pre-Screening and Detailed Scoring	Full-Directional Detailed Scoring	Speedup	Top-5 Retention	False Rejection Rate
Open area	4.196 ms	13.471 ms	18.118 ms	1.345	100%	0
Triangular obstacle	4.123 ms	13.598 ms	18.267 ms	1.342	100%	5%
U-shaped obstacle	4.534 ms	14.296 ms	18.780 ms	1.316	100%	25%

## Data Availability

The original contributions presented in this study are included in the article. Further inquiries can be directed to the corresponding author.

## Supplementary Materials

The following supporting information can be downloaded at: https://www.mdpi.com/article/10.3390/s26041264/s1. Figure S1: Pseudocode of the TPP-CSACO path planning algorithm. Figure S2: Pseudocode of PRM construction and topological progress potential computation. Figure S3: Pseudocode of sector-based direction scoring and selection. Figure S4: Pseudocode of elastic step length with frustration-induced temperature adjustment. Table S1: Friedman test results.
