# Supplementary material for: Topological Progress Potential-Enhanced Continuous-Space Ant Colony Algorithm for Robot Path Planning
Source: Sensors (Basel). 2026 Feb 14;26(4):1264. doi: 10.3390/s26041264 (PMC12943985; doi:10.3390/s26041264)
Supplement: Supplementary file 1 [file sensors-26-01264-s001.zip › sensors-4114595-supplementary.pdf]

Path planning process based on TPP-CSACO

Input: Workspace Ω, obstacle set $\tilde{O}$, start node i_s, goal node i_g;
ACO parameters $(N, M, \text{max\_steps}, \rho_{\text{loc}}, \rho, Q, T_{\text{line}}, R, \dots)$;
PRM/potential parameters (for Algorithm 1); sector scoring parameters (for Algorithm 2);
elastic-step and frustration parameters (for Algorithm 3);
trajectory smoothing parameters $(\alpha, \delta', t_f, \bar{T}, \delta)$;

Output: Final best path P_{best}

Process:

1. Construct signed-distance field (SDF) over Ω from obstacle set $\tilde{O}$ by Eq. (2);
2. $[G, \varphi] \leftarrow$ call Algorithm 1 by Eqs. (5)–(16);
3. Define line-deposition kernel $\tau_p(\cdot; T_{\text{line}}, R)$ by Eqs. (63)–(65);
4. Initialize $\tau(x) \leftarrow \tau_0$; $P_{\text{best}} \leftarrow \emptyset$; $L_{\text{best}} \leftarrow +\infty$;
5. **for** iter $\leftarrow 1$ **to** N **do**
6. SuccessfulPaths $\leftarrow \emptyset$;
7. **for** $k \leftarrow 1$ **to** M **do**
8. $P_k \leftarrow$ continuous path from i_s toward i_g using current $(\tau, \varphi, \text{SDF})$ by repeated calls to Algorithm 2 and Algorithm 3, with step limit max_steps;
9. **if** P_k reaches the goal **then**
10. $L_k \leftarrow$ length of P_k;
11. $\tau \leftarrow$ local pheromone update$(\tau, P_k; \tau_p, T_{\text{line}})$ by Eq. (66);
12. Add (P_k, L_k) to SuccessfulPaths;
13. **end if**
14. **end for**
15. $\tau \leftarrow$ global pheromone update$(\tau, \text{SuccessfulPaths}; \tau_p, Q)$ by Eqs. (67)–(69);
16. **if** SuccessfulPaths $\neq \emptyset$ **then**
17. $[P_{\text{iter}}, L_{\text{iter}}] \leftarrow$ shortest pair in SuccessfulPaths;
18. $P_{\text{iter}}^{\text{opt}} \leftarrow$ trajectory optimization of P_{iter} by pre-smoothing and Bézier rounding using Eqs. (70)–(73);
19. $L_{\text{iter}}^{\text{opt}} \leftarrow$ length of $P_{\text{iter}}^{\text{opt}}$;
20. **if** $L_{\text{iter}}^{\text{opt}} < L_{\text{best}}$ **then**
21. $P_{\text{best}} \leftarrow P_{\text{iter}}^{\text{opt}}$; $L_{\text{best}} \leftarrow L_{\text{iter}}^{\text{opt}}$;
22. **end if**
23. **end if**
24. **end for**
25. **return** P_{best};

Figure S1: Pseudocode of the TPP-CSACO path planning algorithm.

Algorithm 1 PRM construction and Topological Progress Potential

Input: Ω, SDF field, i_s, i_g; N_{sam}, λ; parameters $(K, K_{\text{seg}}, \text{deg}_{\text{min}}, l_c)$; potential parameters (τ)

Output: $G = (V, E)$, $\varphi(v)$

Process:

1. $[N_U, N_B, N_G, N_R] \leftarrow$ per-type U/B/G/R sample counts by Eq. (5);
2. $V_{\text{pre}} \leftarrow$ mixed samples of size $N_U + N_B + N_G + N_R$ including i_s, i_g; $E_{\text{pre}} \leftarrow \emptyset$;
3. **for** each node $P_u \in V_{\text{pre}}$ **do**
4. $[C_{\text{knn}}, C_{\text{sec}}] \leftarrow$ KNN and sector-diverse neighbors of P_u by Eq. (7);
5. Add all collision-free candidate edges (P_u, P_v) from $C_{\text{knn}} \cup C_{\text{sec}}$ to E_{pre} using SDF line-sampling test along segment $P_u P_v$ by Eq. (6), while enforcing deg$(P_u) \geq \text{deg}_{\text{min}}$;
6. **end for**
7. $G_{\text{pre}} \leftarrow (V_{\text{pre}}, E_{\text{pre}})$;
8. $G_s \leftarrow$ grid-based vertex decimation and degree-2 collinearity simplification of G_{pre} with cell size l_c, a fixed collinearity angle of 25°, and edge validation by Eq. (6);
9. **if** i_s and i_g are connected in G_s **then**
10. $G \leftarrow G_s$;
11. **else**
12. $G \leftarrow$ connectivity-repaired graph from G_{pre} using expanding radii R_{set} and candidate edges validated by Eq. (6);
13. **end if**
14. $(V, E) \leftarrow$ node and edge sets of final graph G;
15. **for** each edge $e \in E$ **do**
16. $[W_L(e), W_c(e), W_{Lc}(e)] \leftarrow$ length weight, environment weight and combined weight by Eqs. (8)–(10);
17. **end for**
18. Construct weighted adjacency A, degree matrix D and Laplacian $L = D - A$ from $W_{Lc}(e)$ by Eqs. (11)–(13);
19. $\varphi(i_s) \leftarrow 1$; $\varphi(i_g) \leftarrow 0$; $I \leftarrow V \setminus \{i_s, i_g\}$;
20. Reorder nodes into boundary set $B = \{i_s, i_g\}$ and interior set I;
21. Partition L into blocks $\begin{bmatrix} L_{II} & L_{IB} \\ L_{BI} & L_{BB} \end{bmatrix}$ and solve $L_{II}\varphi_I = -L_{IB}\varphi_B$ for interior potentials φ_I by Eqs. (14)–(15), which ensure harmonicity property Eq. (16);
22. **return** $G = (V, E)$, $\varphi(v)$ for all $v \in V$;

Figure S2: Pseudocode of PRM construction and topological progress potential computation.

Algorithm 2 Sector scoring and direction decision

Input: pos, goal, R, SDF, τ, φ, parameters $(N_\varphi, \eta, N_r, N_\theta, \delta, K_{\text{min}}, \eta_{\text{pre}}, \ldots)$

Output: φ^*, $H_{\text{coa}}(j)$, $r_{\text{safe}}(j,k)$

Process:

1. $[\varphi_j, \omega_j], [r_i, \theta_{j,k}], P_{j,i,k} \leftarrow$ sector geometry around current position,oriented toward goal by Eqs. (17)–(19);
2. **for** $j \leftarrow 1$ **to** N_φ **do**
3.   **for** all cells (i,k) in sector j **do**
4.   allow$(j,i,k) \leftarrow$ safety flag at $P_{j,i,k}$ from SDF by Eq. (20);
5.   **end for**
6. $[f(j,k), M_j, \omega_k, \bar{R}_j] \leftarrow$ first-blocking rings, free-cell set, angular weights and weighted radius by Eqs. (21)–(24);
7. **end for**
8. **for** $j \leftarrow 1$ **to** N_φ **do**
9. $[S_\varphi(j), \tilde{S}_\varphi(j), g(j), B(j)] \leftarrow$ potential-drop, goal alignment and base score by Eqs. (26)–(28);
10. $[\text{cov}_j, \text{dep}_j, \text{qual}_j, G_{\text{geo}}(j), z_{\text{pre}}(j), G_\tau(j)] \leftarrow$ geometric and pheromone factors by Eqs. (29)–(34);
11. $H_{\text{pre}}(j) \leftarrow B(j) \cdot G_{\text{geo}}(j) \cdot G_\tau(j)$ by Eq. (25);
12. **end for**
13. $K_{\text{pre}} \leftarrow \max(K_{\text{min}}, \lceil \eta_{\text{pre}} \cdot N_\varphi \rceil)$; $J_{\text{cand}} \leftarrow$ top K_{pre} indices of $H_{\text{pre}}(j)$ by Eq. (35);
14. **for** all $j \in J_{\text{cand}}$ **do**
15.   **for** each column k in sector j **do**
16. $[d_{\text{safe}}, d_{\text{entry}}, d_{\text{block}}, d_{\text{left}}, d_{\text{right}}] \leftarrow$ five-point SDF samples and 2δ-isoline interpolation $r_{\text{interp}}(j,k)$ by Eqs. (36)–(38);
17.   **end for**
18. $r_{\text{interp}}(j,\cdot) \leftarrow$ lateral consistency correction by Eq. (39);
19. $[r_{\text{final}}(j,k), r_{\text{safe}}(j,k)] \leftarrow$ final and safe radii per column by Eqs. (40)–(41);
20. $\bar{R}_j^{\text{safe}} \leftarrow$ angularly weighted safe radius from $r_{\text{safe}}(j,k)$ using ω_k, with the same weighting as Eqs. (23)–(24);
21. $\xi_j \leftarrow$ normalized penetration depth from $\bar{R}_j^{\text{safe}}$ and R by Eq. (42);
22. $[G_{\text{dir}}(j), T_\tau(j), G_{\text{geom}}(j)] \leftarrow$ direction, pheromone and geometry factors by Eqs. (44)–(52);
23. $H_{\text{coa}}(j) \leftarrow G_{\text{dir}}(j) \cdot T_\tau(j) \cdot G_{\text{geom}}(j)$ by Eq. (43);
24. **end for**
25. $p(j) \leftarrow \text{softmax}(H_{\text{coa}}(j))$ over $j \in J_{\text{cand}}$;
26. $j^* \leftarrow$ random sample from $p(j)$; $\quad \varphi^* \leftarrow \varphi_{j^*}$;
27. **return** $\varphi^*, H_{\text{coa}}(j), r_{\text{safe}}(j,k)$;

Figure S3: Pseudocode of sector-based direction scoring and selection.

Algorithm 3 Elastic step length and frustration induced temperature rise machine

Input: pos, φ_j, goal, R, SDF, state (f, α_t, H, T), constants $(R_{\text{min}}, \lambda', f_{high}, T_0, T_{\text{max}}, q)$

Output: New position pos_new and updated state (f, α_t, H, T)

Process:

$d_{\text{goal}} \leftarrow \|\text{goal} - \text{pos}\|$;
if $d_{\text{goal}} < R$ **then**
$\{d'_{\text{center}}\} \leftarrow$ SDF samples along the ray from pos to goal by Eq. (62);
if $\min_i\{d'_{\text{center}}\} \geq 2\delta$ **and** $\text{SDF(goal)} \geq \delta$ **then**
pos_new $\leftarrow$ goal;
$f \leftarrow \lambda' f$; $\alpha_t \leftarrow 1$; $H \leftarrow 0$; $T \leftarrow T_0$; $I_t \leftarrow 0$;
return pos_new, (f, α_t, H, T);
end if
end if
$R_{\text{safe}} \leftarrow$ safe travel radius in direction φ_j by Eq. (36)-(41);
$R_{\text{nom}} \leftarrow \min(R, R_{\text{safe}})$ by Eq. (56);
$R_{\text{use}} \leftarrow \max(R_{\text{min}}, R_{\text{nom}} \cdot \alpha_t)$ by Eq. (60);
pos_try $\leftarrow$ pos $+ R_{\text{use}} \cdot (\cos \varphi_j, \sin \varphi_j)^{\text{T}}$;
if movement from pos to pos_try is blocked (collision or SDF $< \delta$) **then**
pos_new $\leftarrow$ pos; $I_t \leftarrow 1$;
else
pos_new $\leftarrow$ pos_try; $I_t \leftarrow 0$;
end if
$f \leftarrow \lambda' f + (1 - \lambda') I_t$ by Eq. (57);
$\alpha_t \leftarrow 0.5$ **if** $I_t = 1$ **else** 1 by Eq. (58);
$H \leftarrow H + 1$ **if** $f > \theta_h$ **else** 0 by Eq. (61);
if $H \geq 6$ **then**
terminate current ant by Eq. (61);
end if
$T \leftarrow T_0 + (T_{\text{max}} - T_0) f^q$ **if** $f > 0.3$ **else** T_0 by Eq. (59);
return pos_new, (f, α_t, H, T);

Figure S4: Pseudocode of elastic step length with frustration-induced temperature adjustment.

Table S1: Friedman test results.

Metric	10×10 map			20×20 map			50×50 map		
	χ^2	df	p-value	χ^2	df	p-value	χ^2	df	p-value
**Path Length**	182.74	7	<0.001	87.44	7	<0.001	24.68	8	<0.002
**Total Turn**	200.56	7	<0.001	96.85	7	<0.001	30.53	8	<0.001
**Max Turn**	168.92	7	<0.001	84.80	7	<0.001	27.73	8	<0.001
**Viol Percent**	175.75	7	<0.001	89.84	7	<0.001	21.31	8	<0.001
J_{eq}	202.48	7	<0.001	92.96	7	<0.001	21.73	8	<0.001
